# Efficacy of green synthesized silver nanoparticles via ginger rhizome extract against *Leishmania major* in vitro

**DOI:** 10.1371/journal.pone.0255571

**Published:** 2021-08-18

**Authors:** Mohsen Mohammadi, Leila Zaki, Amir KarimiPourSaryazdi, Pooya Tavakoli, Atiyeh Tavajjohi, Reza Poursalehi, Hamid Delavari, Fatemeh Ghaffarifar

**Affiliations:** 1 Department of Materials Engineering, Nanomaterials Group, Tarbiat Modares University, Tehran, Iran; 2 Department of Parasitology, Faculty of Medical Sciences, Tarbiat Modares University, Tehran, Iran; National Centre For Cell Science, INDIA

## Abstract

**Introduction:**

Leishmaniasis is a major public health problem that causes by parasite of the genus *Leishmania*. The pentavalent antimonial compounds that used for treatment are not safe or effective enough. The aim of the present study was preparation and evaluation of the efficacy of green synthesized silver nanoparticles against *Leishmania major* (*L*. *major*) in vitro.

**Methods:**

To synthesis silver (Ag) nanoparticles (NPs), ginger extract was added to the 0.2mM AgNO_3_ aqueous solution (1:20). Effects of different concentrations of Ag-NPs on the number of *L*. *major* promastigotes were investigated using counting assay. The MTT test was applied to determine the toxicity of Ag-NPs on promastigotes of *L*. *major*, as well as, macrophage cells. Then, to evaluate the anti-amastigotes effects of Ag-NPs, parasites within the macrophages were counted by light microscope. Furthermore, to determine the induced apoptosis and necrotic effects of Ag-NPs on promastigotes, flow cytometry method was employed using annexin staining.

**Results:**

The effect of Ag-NPs on promastigotes and amastigotes of *L*. *major* was effective and has a reverse relationship with its concentration. According to the results of anti-amastigote assay, the IC50 value of this nanoparticle was estimated 2.35 ppm after 72h. Also, Ag-NPs caused Programmed Cell Death (PCD) in promastigotes of *L*. *major* and showed 60.18% of apoptosis.

**Discussion:**

Based on the mentioned results, it can be concluded that Ag NPs has a beneficial effect on promastigote and amastigote forms of *L*. *major* in vitro. Hence, these nanoparticles could be applied as promising antileishmanial agents for treatment of Leishmania infections.

## Introduction

Leishmaniasis is still one of the *major* global health problems that causes by parasite of the genus Leishmania and can be transmitted through the bite of female phlebotomine sandflies in tropical and subtropical climates regions [1, 2]. The poorest are at the highest risk of morbidity and mortality in five continents of the World. Due to Leishmania parasites Clinical manifestations occur in at least three different forms, such as cutaneous leishmaniasis (CL), mucocutaneous leishmaniasis (MCL), and visceral leishmaniasis (VL). Based on the diverse clinical forms of cutaneous leishmaniasis, the consequent complications will be different ranging from self-limited skin disease to diffuse (severe) visceral disease [3, 4]. On average, approximately 350 million people are estimated at risk of leishmaniasis and 12 million people have become infected throughout the world. Noteworthy, this skin infection is prevalent in 98 countries. Every year, there are more than 1 million new cases of cutaneous leishmaniasis and 500,000 new cases of visceral leishmaniasis among people in developed and developing countries, according WHO reports [5, 6]. Despite efforts of scientists, there is no effective prevention and treatment strategy against leishmaniasis. Over the last 70 years, chemotherapy has still remained the most popular treatment for leishmaniasis diseases, as well as, antimony based drugs, such as sodium stibogluconate (Pentostam) and meglumine antimoniate (Glucantime) have affected the highest percentage of cures [7–9]. Also, it has been demonstrated that these medications are able to prevent the production of adenosine triphosphate (ATP) via interruption in phosphokinase enzyme activity [9]. Accumulating evidence has shown that drugs used for leishmaniasis have not been completely successful yet, and also have several disadvantages such as low sensitivity, non-specific, toxicity, financial burden, long-term treatment, drug and parasite resistance, painful administration route, treatment failures, as well as damaging some tissues [9–11]. Since antiparasitic drugs have unwilling side effects and also may cause serious problems, discovering and development of appropriate treatment methods or potent anti-Leishmania compounds are needed [12]. Currently, the medical application of nanoparticles (NPs) [13–16] is an exciting innovation that complements the global health community’s efforts to end the leishmaniasis endemics. For instance, chitosan, Au, Ag, Fe_3_O_4_, TiO_2_ and ZnO were employed to treat the diseases are related *L*. *major* [17, 18]. Moreover, some of the plants extracts like Aloe-emodin, Artemisinin, Thymus migricus and Tussilago farfara were used to remedy leishmania parasite [19–21]. The previous researches have been shown that Silver nanoparticles have antileishmanial effects [22–25].

In this research, based on proved antimicrobial behavior of Ag NPs [26–29], as well as according to antileishmania activity of silver [30], as well as antimicrobial nature of ginger extract [31], the silver NPs synthesized via ginger extract to evaluate the therapeutic effects against *L*. *major* for the first time according to the best of our knowledge. In this respect, silver NPs prepared in a green route through ginger extract initially, followed by characterizing via UV-visible spectroscopy and TEM images.

## Materials and methods

### Extract preparation and synthesis of silver nanoparticles

In this study, extracts and silver nanoparticles prepared according to reported investigation on green synthesis of silver nanoparticles [19]. In this regard, in the first step, ginger rhizome was washed by deionized (DI) water, followed by slicing to the fine pieces, after which exposed to a shadow at 27°C for four days to become completely dry. Afterward, 0.2 g of fine gingers was poured into 100 mL DI water, stirring at 80°C for 40 minutes. Furthermore, the extract was filtered by Whatman No.1 paper and centrifuged for 5 minutes at 4000 rpm, before passing through a 0.22 syringe filter, and maintained at 4°C far from the light. To synthesis silver NPs, the 0.2mM AgNO_3_ solution prepared initially, after which the extract was putted on the AgNO_3_ aqueous solution (1:20). First, the solution was approximately colorless and by progression of the reaction, the color changed from the light yellow to dark brown, which could be a visual attest to confirm formation of silver NPs. Next, by employing a 12 kD dialysis bag, the silver NPs dialyzed in water for 24 h, and filtered via 0.22μm syringe filters.

### Characterization of Ag NPs

In the first step of characterization, a SPUV-26 SC-Tech spectrophotometer was employed to carry out the UV–visible spectroscopy to assess formation of green synthesized silver NPs. Moreover, to evaluate the size and shape of nanoparticles, Transmission electron microscopy (TEM) method was carried out via Leo 912 AB microscope.

### Collection and cultivation of parasites

In this work, *L*. *major* Iranian standard strain promastigotes (MRHO/IR/75/ER) were obtained from parasitology department of Tarbiat Modares University. For growth and replication of the *L*. *major* promastigotes the nutrient RPMI 1640 medium enriched with penicillin (100 unit ml−1), streptomycin (100 μgml−1) fetal calf serum (10% v/v) were used and after that incubated in 25 ± 1°C.

### Culturing of macrophage cells

The study used macrophages were derived from RAW.264.7 macrophage cell line which is obtained by the Parasitology Department at Tarbiat Modares University of Tehran, Iran. At the beginning, RAW.264.7 macrophages were cultured in RPMI 1640 containing 10% FBS (Gibco-BRL, France), penicillin (100 unit ml^−1^), and streptomycin (100 μgml^−1^) (Sigma Chemical Co.), then these culture flasks incubated in a humidified atmosphere of 95% air and 5% CO_2_ at 37°C.

### Promastigote assay

For this assay, 100 μL of promastigotes (2 × 10^6^ cell/mL) was seeded in 96-well plate containing 100 μL of RPMI1640 pluse 15% FBS in the presence of several concentrations (40, 20, 10, 5, 2.5, 1.25, 0.625 and 0.312 ppm) of the Ag NPs solution as triplicate and were kept at a temperature of 24 ± 2°C for 24, 48, and 72 hours. In addition, AmpB (GILEAD UK) (1 μg/mL) and Glucantime (Sanofi-Aventis France) (100 μg/mL) were used as positive control groups [32]. At the end, the direct counting method by hemocytometer chamber (Neubauer chamber) was performed to evaluate the anti-leishmanial effects of Ag NPs on promastigotes of *L*. *major* and the results compared with control groups as well as analyzed by Graph pad Prism 5.0 [16].

### Cytotoxic effects of green synthesized silver nanoparticles on *L*. *major* promastigotes

In order to assessing the antileishmanial effects of silver NPs upon the promastigotes of *L*. *major* parasites, 3- (4, 5-dimethylthiazol- 2‑yl) -2, 5‑diphenyl‑tetrazolium bromide (MTT, Sigma‑Aldrich) assay was taken. Briefly, in 96-well microtiter plates *L*. *major* promastigotes (100 μl, 1×105 cell ml−1) were cultivated in the presence of different amounts of silver NPs (40 to 0.16 ppm) for 24, 48, and 72 h at 26°C. After that, 20 μL of MTT reagent (5 mg/mL) was added to the wells and further incubated for 3–5 hours in the incubator. Subsequently, they centrifuged for 10 minutes at 3000 rpm. For observation of formazan crystals the contents of each well was removed and replaced with 100 μL of dimethyl sulfoxide (DMSO) and then kept at room temperature for 30 minutes. Eventually, using an ELISA plate reader (ELX800), absorbances were read within 30 minutes at a wavelength of 570 nm and the results analyzed by GraphPad prism5 software to determine IC50 of Leishmania growth. The percentage of viability was measured using the following formula:

$$Cell\;Viability\;(\%)=\frac{\left(Drug\;well\;absorption)–(Blank\;well\;absorption\right)}{\left(Control\;well\;absorption)–(Blank\;well\;absorption\right)}\times 100$$

### Cytotoxic effects of green synthesized silver nanoparticles on macrophages

In the present study, for estimation of uninfected macrophage viability, RAW264.7 macrophages which were grown and propagated in complete RPMI1640 containing 10% FBS were harvested and cultured in 96-well culture plates (100 μL, 1×10^5^ cell mL^−1^) with different concentrations of silver NPs (40 to 0.16 ppm). The samples were placed in the incubator for three days at 37°C in a 5% CO_2_. After incubation period, MTT assay was performed as mentioned above.

### Amastigote assay

At the beginning, Murine macrophage-derived RAW 264.7 cells(1×10^5^ cell ml−1) were seeded into the 12-well micro-plates with rounded coverslips on the bottom, then the samples were subsequently incubated overnight at 37°C in a CO_2_ incubator (5% CO_2_ and 95% relative humidity). In this context, non-adherent macrophages were omitted thoroughly with the use of cold phosphate buffer saline (PBS) whereas adherent macrophages were infected through the stationary phase of *L*. *major* Promastigotes (at 10:1 ratio of parasites/macrophages). Following incubation for 24 h at 37°C with 5% CO_2_, the plate was again washed (as above) and excess cells were eliminated. Then, infected macrophages were treated with different volumes of silver NPs (1.25 and 2.5 ppm) and were incubated in a humidified atmosphere of 95% air and 5% CO_2_ at 37°C. Moreover, the infected cells in the absence of the drug were employed as a reference of negative control groups. After 24 h incubation, the supernatant was discarded and the coverslips were fixed in methanol to stain with Giemsa solution. Tachyzoites within the macrophages was counted and the results were compared with the untreated control group by using an optical microscope [16].

### Flow cytometry analysis

To identify the drug-induced apoptosis and necrosis Annexin-V FLUOS staining kit (Bio-vision, USA) with annexin V-FITC and PI (propidium iodide) staining was used. In brief, 2×10^6^ ml−1 promastigotes were exposed to 5 μg/ mL concentration of Ag NPs and placed in the incubator, as mentioned above. In addition, *L*. *major* promastigotes without drug were considered as the control group. 24 hours later, the samples harvested and centrifuged at 3000 rpm for 10 min. After emptying the supernatant liquid, 500μL binding buffer, 5μL annexin V and 5μL propidium iodide (PI) were added to the pellets and incubated at 25°C under dark conditions for 5 min. Absorption of annexin-v by cells was measured by FACSCaliber (FACSCanto II) and the percentage of apoptotic, necrotic and normal cells for each sample were analyzed using FlowJo Software [33].

### Statistical analysis

SPSS software version 21 (SPSS Inc., Chicago, IL, USA) and Graph Pad Prism version 5.0 (GraphPad Software, Inc., San Diego, CA) were applied in order to statistical analysis. All the obtained results were expressed as mean ± S.D and compared by one-way ANOVA as parametric tests. Additionally, a probability (P) value less than 0.05 (p < 0.05) was regarded as the statistical differences.

## Results

### UV-visible spectroscopy of silver NPs

**Fig 1:**
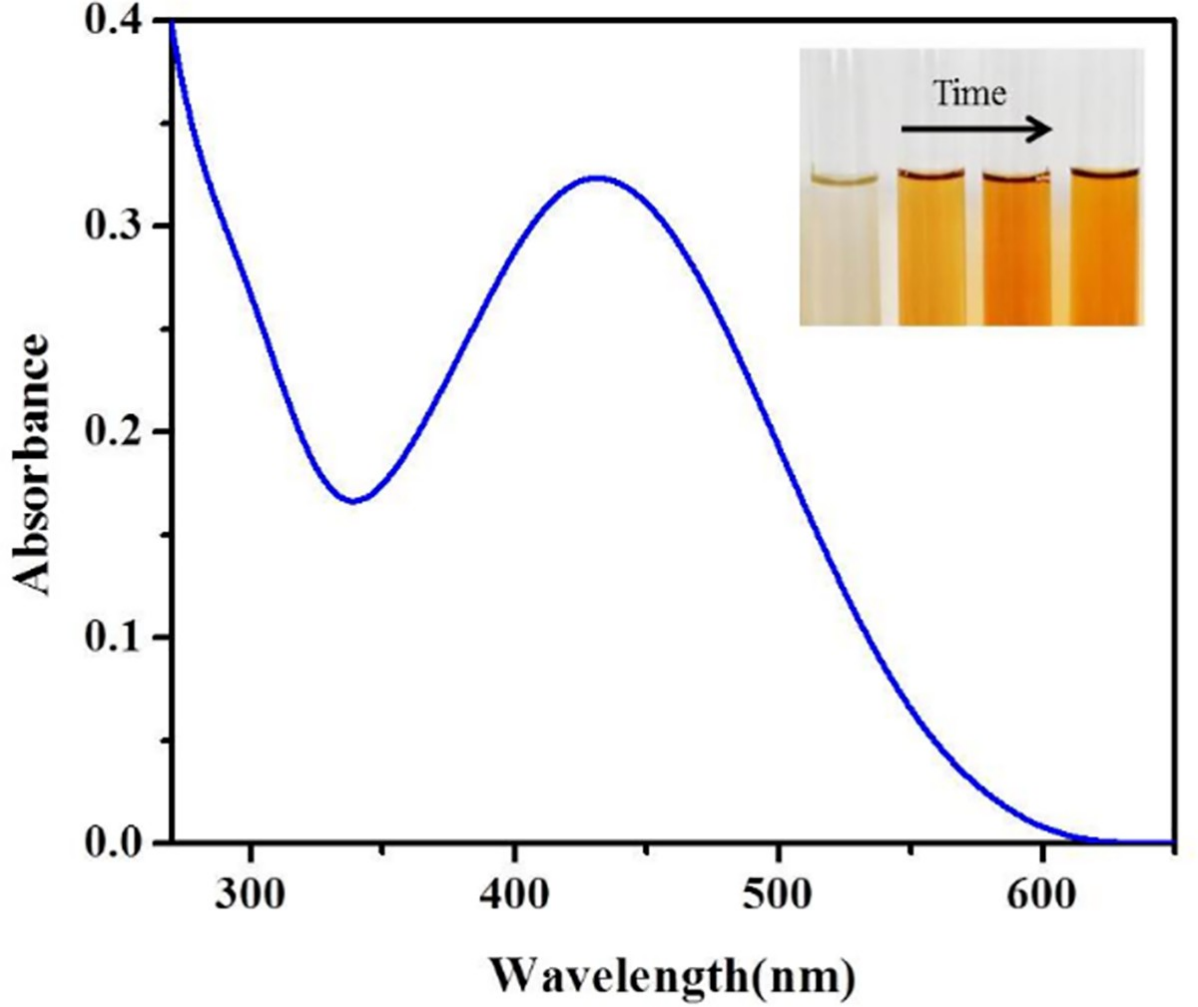
The UV-visible spectroscopy of green synthesized silver NPs via ginger extract.

To evaluate the formation of silver NPs, UV-visible spectroscopy of green synthesized solution was carried out against water and the spectrum is shown in Fig 1. The diagram shows a peak between 400 and 460 nm (430 nm), which is the surface plasmon resonance (SPR) of green synthesized silver NPs and confirms formation of silver NPs. The figure illustrate that by increasing the time of reaction, SPR intensity elevates, which it means the formation of higher amounts of silver NPs.

## TEM image of silver NPs

**Fig 2:**
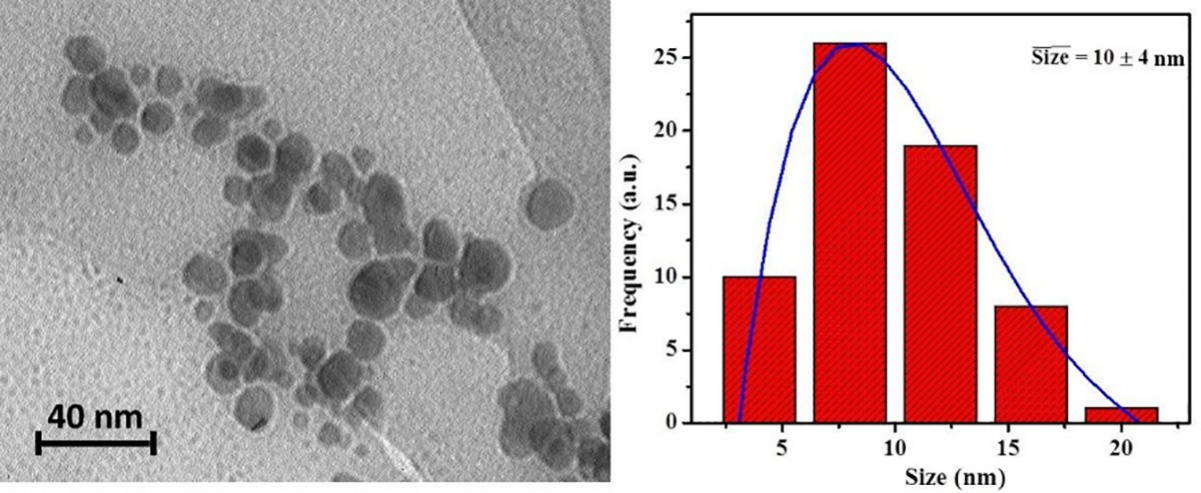
The TEM image (a) and size distribution (b) of green synthesized silver NPs via ginger extract.

The shape and size of green synthesized silver NPs is shown in Fig 2A via TEM image. Moreover, the size distribution of NPs is presented in Fig 2B. As it can be seen, the TEM image shows that the NPs are approximately spherical and the mean size of silver NPs is 10 ± 4 nm.

### The effect of silver NPs on promastigotes growth

**Fig 3:**
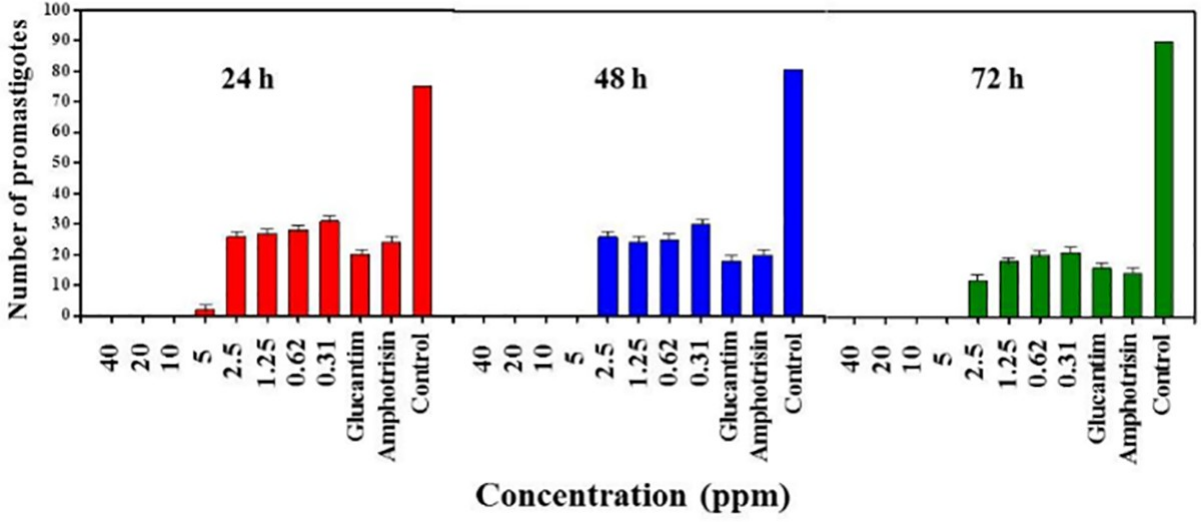
Mean and standard deviation of the number of promastigotes of *L*. *major* (×10^4^) cultured with different concentrations of silver NPs in comparison with control groups for 24, 48 and 72h.

The effects of different concentrations of Ag NPs on the number of *L*. *major* promastigotes were investigated after incubation for 24, 48, and 72 h (Fig 3). In this test, considerable differences were observed between all concentrations of Ag NPs and control groups (p < 0.05). The results showed that by increasing the concentration of Ag NPs, proliferation of *L*. *major* promastigotes decreased remarkably. The concentrations of 40, 20, 10, and 5 ppm of Ag NPs with incubation times of 24, 48, and 72 h showed the most efficacies, whereas the concentration of 0.312 and 0.39 ppm after 24 and 48 h incubation, respectively, presented the least efficacies on inhibiting the proliferation and mobility of *L*. *major* promastigotes. Furthermore, it was found that high concentrations (40, 20, 10 and 5 ppm) of Ag NPs completely inhibit the proliferation of *L*. *major* promastigotes after 24, 48, and 72 h.

### The cytotoxicity of silver NPs to the promastigotes by MTT

**Fig 4:**
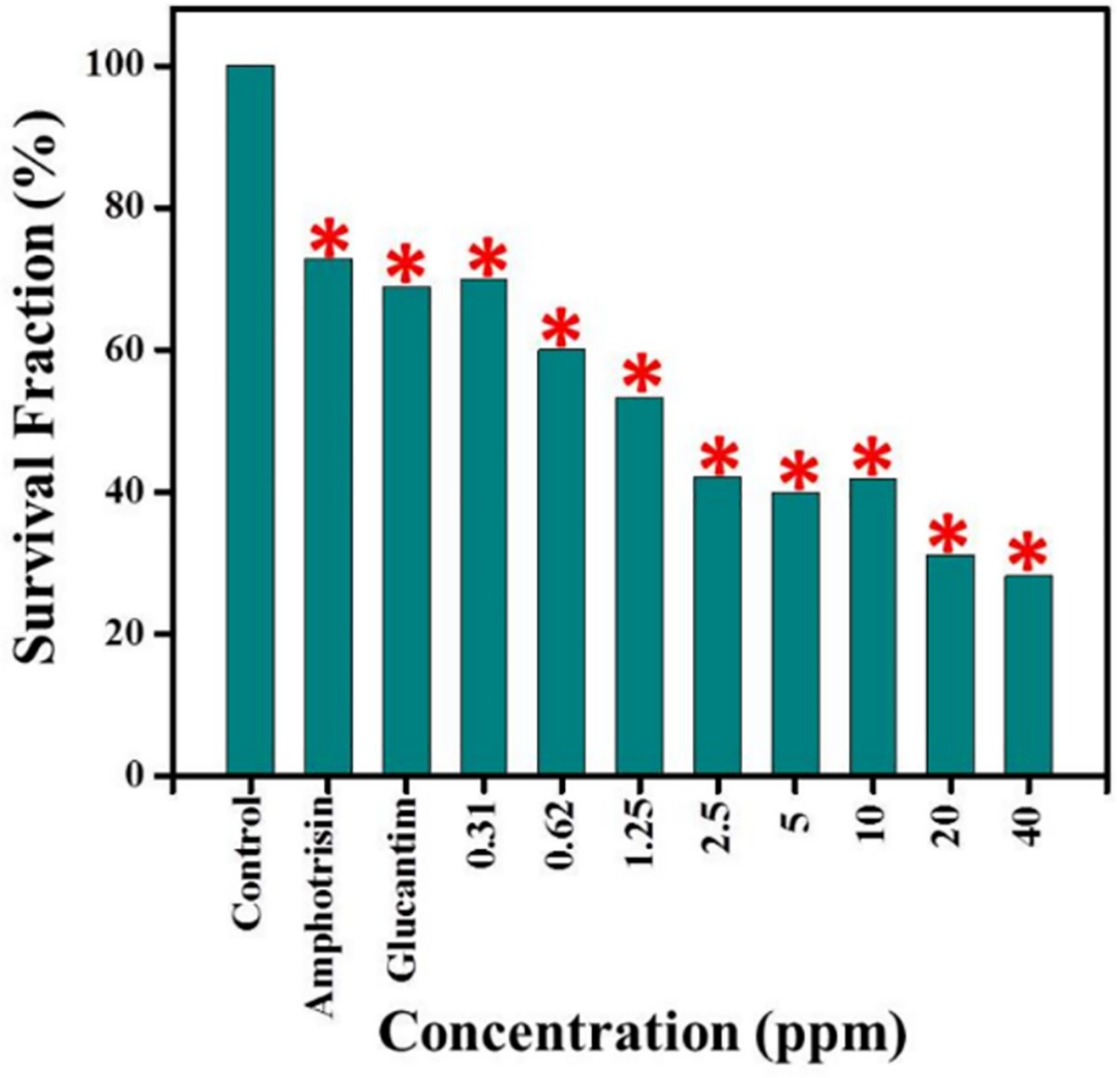
The viability of *L*. *major* promastigotes in the presence of different concentrations of the Ag NPs (40 to 0.16 ppm), after 72 h incubation and compared them with the control groups.

Cytotoxicity effect of the silver NPs against *L*. *major* promastigotes was investigated by optical density (OD) following MTT assay. As Fig 4 shows, parasite viability was found to be based on a dose-dependent response and decreased by increasing the silver NPs concentration. In other words, the maximum toxicity was related to concentration of 40 ppm after 24 h, whereas at concentration of 3.12 ppm of the silver NPs the results were close to the reference drug (AmpB).

### The cytotoxicity of silver NPs to macrophages by MTT

**Fig 5:**
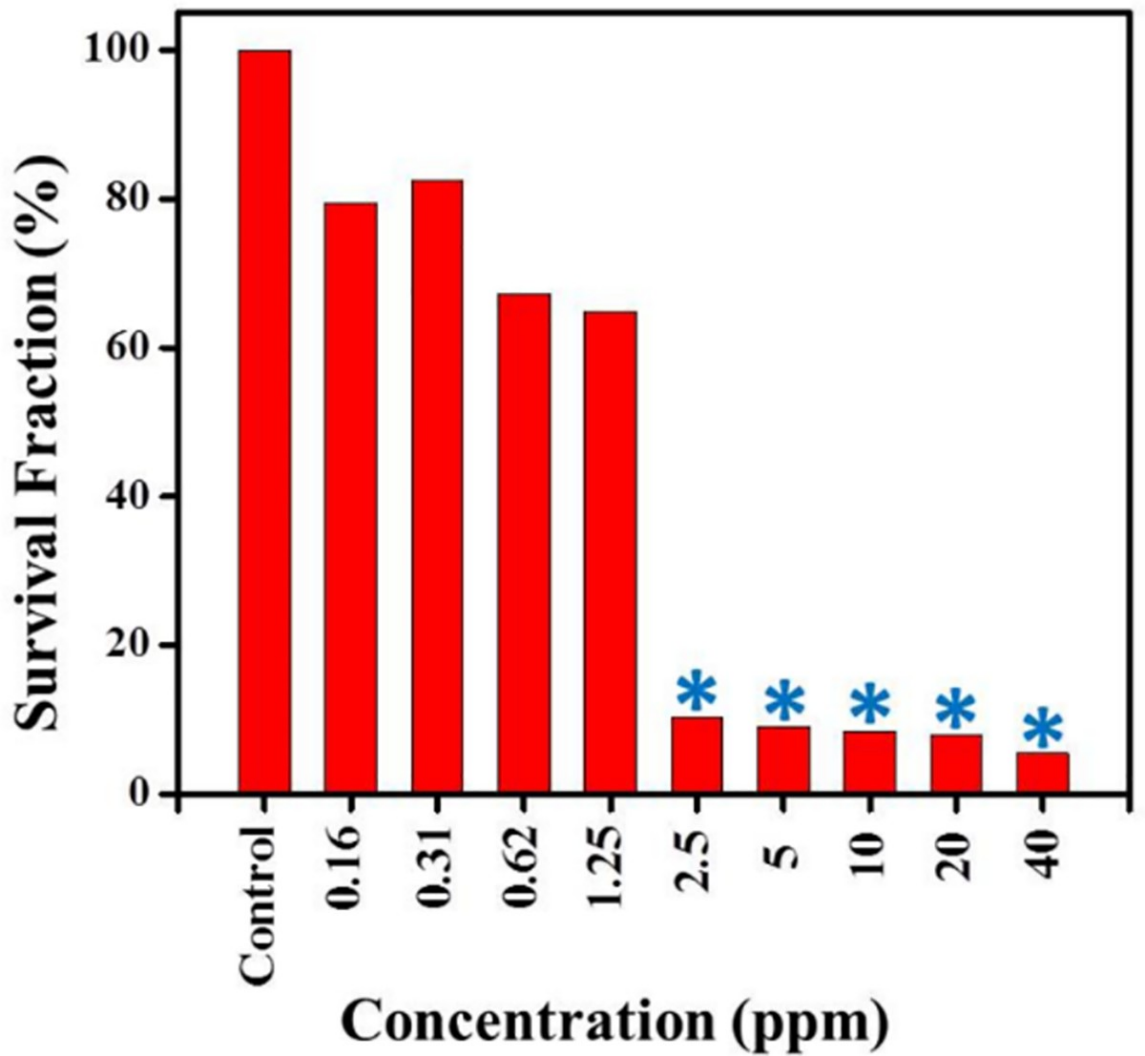
Percentage of viability of the uninfected RAW macrophages with different concentrations of the Ag NPs (40 to 0.16 ppm), after 72 h incubation in comparison to control group.

It was observed that high concentrations of the silver NPs have more toxic impacts upon macrophage cells than low concentrations compared to the control group (Fig 5).

### Anti-amastigote assay

In the current investigation, the anti-amastigote effect of the silver NPs on infected macrophage cells was determined. In this regard, the mean number of amastigotes/macrophage was examined by optical microscope after 72 h of incubation. The percentage of infectivity in the infected macrophages were 14.75 and 27.5 after being treated with 1.25 and 2.5 ppm of silver NPs, respectively, while in the negative control group, 30% of macrophages were infected with amastigotes (Table 1). Additionally, the IC50 value of the silver NPs on macrophage cells was equal to 2.35 ppm for a time period of 72 h.

**Table 1:** Mean and standard deviation of amastigotes per macrophage and percentage of infected macrophages for treated and control groups.

Groups	Number of amastigote in each macrophage (M±SD)	Percentage of infected macrophages
Silver NPs (1.25 ppm)	1.48±0.04	14.75%
Silver NPs (2.5 ppm)	1.36±0.04	27.5%
Control untreated	2.53±0.5	30%

### Flow cytometry assay

In order to calculate the percentage of necrotic, apoptotic, and alive cells in *L*. *major* promastigotes flow cytometry assay was employed after staining with Annexin-V and PI.

**Fig 6:**
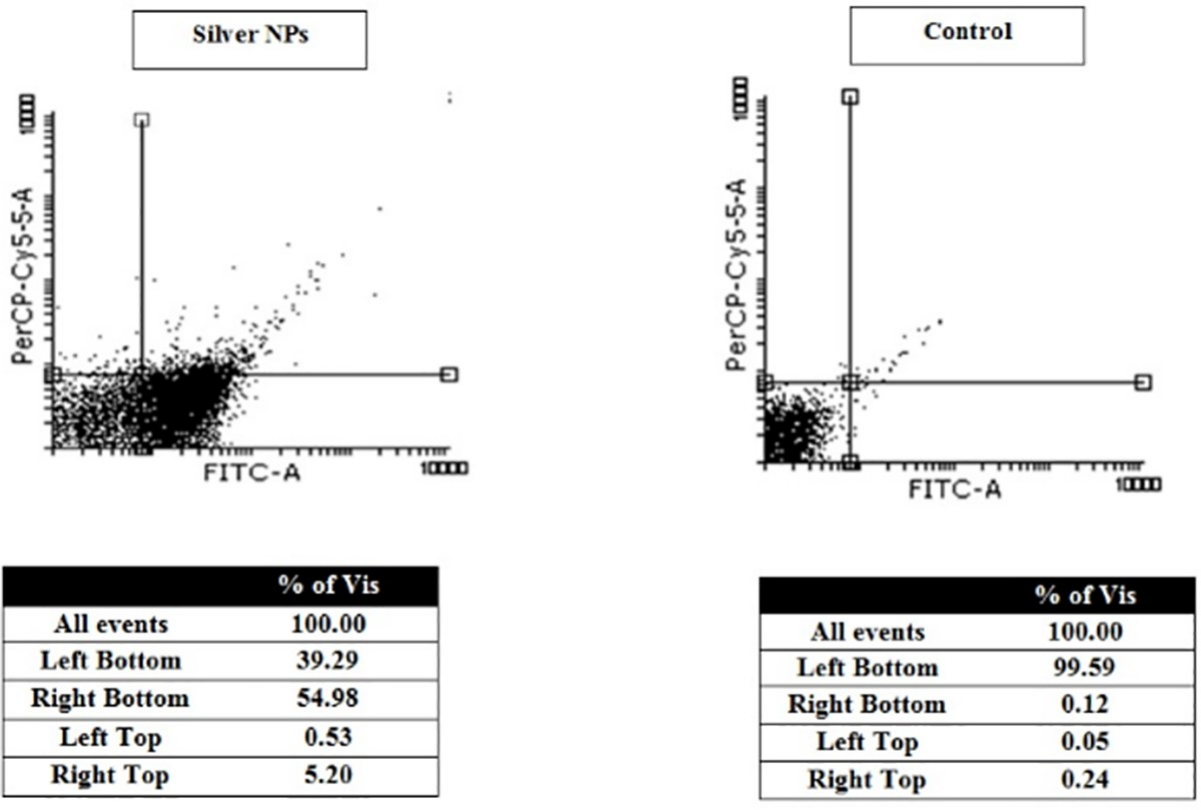
Flow cytometry results of the effect of Silver NPs on promastigotes of *L*. *major* and comparing with the control group (untreated) after 24 h. Regions of quardrate show necrosis promastigotes in left top, late apoptosis in Right top, Right bottom region belongs to apoptotic promastigotes and left bottom region belongs to live promastigotes.

Here, this study showed that the percentage of apoptosis and necrosis induced in promastigotes exposed to 5 ppm concentrations of silver NPs after 72 h incubation were estimated 60.18% and 0.53%, respectively. However, the percentage of alive cells in the control group (without treatment) was determined 99.59%. The outputs of flow cytometry analysis are represented in Fig 6.

## Discussion

Leishmaniasis is now widely remained as one of the main health problems all around the world. Current therapy against leishmaniasis wasn’t satisfactory, and applied drugs (glucantime and pentostam) are not the complete options, because of many side effects, expensive cost, unacceptable toxicity, painful administration, as well as, the appearance of drug resistance in some endemic areas were limited. Accordingly, researchers are continuously seeking to develop and design effective anti-leishmanial agents that could fulfil entirely the treatment goals. In previous studies, the results showed that silver nanoparticles have anti-leishmanial effect [22–25]. Mohebali et al. found with 100 ppm of Nanosilver could damaged 85% of infected macrophages with amastigotes of *L*. *major* [25]. In the present study, the effectiveness of various concentrations of green synthesized silver nanoparticles via ginger rhizome extract against *L*. *major* promastigotes, as well as amastigotes was investigated. Considerable toxicity has been observed for amastigotes and promastigotes of *L*. *major* in the presence of the NPs. The findings showed that proliferation of *L*. *major* promastigotes significantly decreased by increasing the Ag-NPs concentration and exposure time compared to standard drugs, Amphotericin B and Glucantim. In other words, high concentrations (40, 20, 10 and 5 ppm) of Ag NPs completely inhibited the proliferation of *L*. *major* promastigotes after 24, 48, and 72 h. The current MTT assay results displayed that the percentage viability of macrophage cells and *L*. *major* promastigotes exposed to NPs decreased in a concentration-dependent manner, compared to the control group. The viability percentages for macrophages and *L*. *major* promastigotes treated with the maximum concentration of NPs (40 ppm) was reported 7.3% and 32.2%, respectively. The mean number of amastigotes in each macrophage was decreased by 1.25 and 2.5 ppm of Ag-NPs after 72 h of incubation compared with control groups. In addition, the IC50 value against this parasitic form of *L*. *major* was 2.35 ppm after 72 h exposure. Therefore, it found that these nanoparticles induced the inhibition of the proliferation rate of intramacrophage amastigotes and also can be effective in reducing infected macrophages. As reported in previous studies, silver nanoparticles are able to induce programmed cell death in different cells [34–36]. Also, our findings showed that regarding to control group (promastigotes without treatment), induction of apoptosis was increased in promastigotes of *L*. *major* after exposure to 5 ppm nanoparticles, and hence these apoptotic effects were appreciable. Therefore, it is inferred that Ag-NPs could be effective against *L*. *major* by inducing apoptosis. Many studies reported the positive effects of silver nanocomposites into different microorganisms and some diseases [37]. In this regard, cytotoxic effect of these nanocomposites against bacteria, viruses, fungi and different types of cancer has been examined, and results demonstrated that silver nanocomposites has satisfactory levels of cytotoxicity effect against these cancers and microorganisms [17, 37, 38]. Although a number of studies have demonstrated the antimicrobial properties of silver nanocomposites in some parts of the world, more investigations are needed to better understand of anti-parasitic activities of these nanomaterials against leishmania parasite. In a study conducted by Mohebali and colleagues, the antileishmania activities of silver nanoparticles on *L*. *major* were investigated in vitro and in vivo conditions. According to their results, silver nanoparticles reduced proliferation of amastigote stages of *L*. *major* same as the reference drug. Also, they concluded that nano-silver can be useful to prevent secondary infection in cutaneous leishmaniasis caused by *L*. *major* [25].

In another study, the effect of silver (Ag) NPs at the presence of ultraviolet (UV) light led to a reduction in the metabolic activity and proliferation rates of *Leishmania tropica* [23].

In the Jebali and Kazemi (2013) study, they assessed antileishmanial effects of five nanoparticles including (ZnO NPs, Au NPs, TiO_2_ NPs and etc.) on *L*. *major* under invisible ultraviolet(UV), infrared light(IR) and dark conditions. Results revealed that all of the nanoparticles had inhibitory effects on *L*. *major* parasite, but the greatest antileishmanial activity was related to silver (Ag) NPs during illumination [17]. Based on the above mentioned points, it can be concluded that silver (Ag) NPs can be applied as promising antileishmanial agents for treatment of Leishmania infections.

## Conclusion

Recently, the use of nanoparticles has continuously increased because of their unique structural characteristics for the treatment of various diseases. The results in this study indicated acceptable level of in vitro activity of silver (Ag) NPs against *L*. *major* promastigotes as well as intracellular amastigotes. Also, the results of flow cytometry demonstrated that (Ag) NPs can cause Programmed Cell Death (PCD) in promastigotes of *L*. *major* and showed 60.18% of apoptosis in the exposed group of promastigotes. However, the research suggests that further efforts should be done in order to identification of antileishmanial activities of these nanoparticles under in vivo condition.

## Supporting information

S1 Data (DOCX)

S2 Data (DOCX)

S3 Data (DOCX)

S4 Data (DOCX)

## Abbreviations

Ag: silver
ATP: Adenosine Triphosphate
CL: Cutaneous Leishmaniasis
MCL: Mucocutaneous Leishmaniasis
MTT: 3- (4, 5-dimethylthiazol- 2‑yl) -2, 5‑diphenyl‑tetrazolium bromide
NPs: Nanoparticles
PCD: Programmed Cell Death
P value: Probability value
SD: Standard Deviation
SPR: Surface Plasmon Resonance
TEM: Transmission electron microscopy
VL: Visceral Leishmaniasis
UV: Ultraviolet
