# Supplementary material for: Efficacy of green synthesized silver nanoparticles via ginger rhizome extract against Leishmania major in vitro
Source: PLoS One. 2021 Aug 18;16(8):e0255571. doi: 10.1371/journal.pone.0255571 (PMC8372886; doi:10.1371/journal.pone.0255571)
Supplement: S1 Data — (DOCX) [file pone.0255571.s001.docx]

AG 80

Amastigote Assay

| **Descriptives** | | | | | | | | |
| --- | --- | --- | --- | --- | --- | --- | --- | --- |
| VAR00002 | | | | | | | | |
|  | N | Mean | Std. Deviation | Std. Error | 95% Confidence Interval for Mean | | Minimum | Maximum |
|  |  |  |  |  | Lower Bound | Upper Bound |  |  |
| .00 | 2 | 2.5350 | .51619 | .36500 | -2.1028 | 7.1728 | 2.17 | 2.90 |
| 1.25 | 2 | 1.4550 | .00707 | .00500 | 1.3915 | 1.5185 | 1.45 | 1.46 |
| 2.50 | 2 | 1.3750 | .00707 | .00500 | 1.3115 | 1.4385 | 1.37 | 1.38 |
| Total | 6 | 1.7883 | .62378 | .25466 | 1.1337 | 2.4429 | 1.37 | 2.90 |

| **Multiple Comparisons** | | | | | | |
| --- | --- | --- | --- | --- | --- | --- |
| Dependent Variable: VAR00002 | | | | | | |
| Tukey HSD | | | | | | |
| (I) VAR00001 | (J) VAR00001 | Mean Difference (I-J) | Std. Error | Sig. | 95% Confidence Interval | |
|  |  |  |  |  | Lower Bound | Upper Bound |
| .00 | 1.25 | 1.08000 | .29808 | .072 | -.1656 | 2.3256 |
|  | 2.50 | 1.16000 | .29808 | .060 | -.0856 | 2.4056 |
| 1.25 | .00 | -1.08000 | .29808 | .072 | -2.3256 | .1656 |
|  | 2.50 | .08000 | .29808 | .962 | -1.1656 | 1.3256 |
| 2.50 | .00 | -1.16000 | .29808 | .060 | -2.4056 | .0856 |
|  | 1.25 | -.08000 | .29808 | .962 | -1.3256 | 1.1656 |

AG 100 AMASTI

| **Descriptives** | | | | | | | | |
| --- | --- | --- | --- | --- | --- | --- | --- | --- |
| VAR00002 | | | | | | | | |
|  | N | Mean | Std. Deviation | Std. Error | 95% Confidence Interval for Mean | | Minimum | Maximum |
|  |  |  |  |  | Lower Bound | Upper Bound |  |  |
| .00 | 2 | 2.5350 | .51619 | .36500 | -2.1028 | 7.1728 | 2.17 | 2.90 |
| 1.25 | 2 | 1.7000 | .01414 | .01000 | 1.5729 | 1.8271 | 1.69 | 1.71 |
| 2.50 | 2 | 1.4850 | .00707 | .00500 | 1.4215 | 1.5485 | 1.48 | 1.49 |
| Total | 6 | 1.9067 | .54724 | .22341 | 1.3324 | 2.4810 | 1.48 | 2.90 |

| **Multiple Comparisons** | | | | | | |
| --- | --- | --- | --- | --- | --- | --- |
| Dependent Variable: VAR00002 | | | | | | |
| Tukey HSD | | | | | | |
| (I) VAR00001 | (J) VAR00001 | Mean Difference (I-J) | Std. Error | Sig. | 95% Confidence Interval | |
|  |  |  |  |  | Lower Bound | Upper Bound |
| .00 | 1.25 | .83500 | .29816 | .132 | -.4109 | 2.0809 |
|  | 2.50 | 1.05000 | .29816 | .077 | -.1959 | 2.2959 |
| 1.25 | .00 | -.83500 | .29816 | .132 | -2.0809 | .4109 |
|  | 2.50 | .21500 | .29816 | .770 | -1.0309 | 1.4609 |
| 2.50 | .00 | -1.05000 | .29816 | .077 | -2.2959 | .1959 |
|  | 1.25 | -.21500 | .29816 | .770 | -1.4609 | 1.0309 |
