# Supplementary material for: Efficacy of green synthesized silver nanoparticles via ginger rhizome extract against Leishmania major in vitro
Source: PLoS One. 2021 Aug 18;16(8):e0255571. doi: 10.1371/journal.pone.0255571 (PMC8372886; doi:10.1371/journal.pone.0255571)
Supplement: S2 Data — (DOCX) [file pone.0255571.s002.docx]

MTT Assay for macrophages

Number 1: Control (witout treatment)

Number 2: treated with 40 µg/ml of nanoparticle

Number 3: treated with 20 µg/ml of nanoparticle

Number 4: treated with 10 µg/ml of nanoparticle

Number 5: treated with 5 µg/ml of nanoparticle

Number 6: treated with 2.5 µg/ml of nanoparticle

Number 7: treated with 1.25 µg/ml of nanoparticle

Number 8: treated with 0.625 µg/ml of nanoparticle

Number 9: treated with 0.312 µg/ml of nanoparticle

Number 10: treated with 0.156 µg/ml of nanoparticle

| **Descriptives** | | | | | | | | |
| --- | --- | --- | --- | --- | --- | --- | --- | --- |
| VAR00002 | | | | | | | | |
|  | N | Mean | Std. Deviation | Std. Error | 95% Confidence Interval for Mean | | Minimum | Maximum |
|  |  |  |  |  | Lower Bound | Upper Bound |  |  |
| 1.00 | 2 | .8250 | .03536 | .02500 | .5073 | 1.1427 | .80 | .85 |
| 2.00 | 2 | .0450 | .00707 | .00500 | -.0185 | .1085 | .04 | .05 |
| 3.00 | 2 | .0650 | .00707 | .00500 | .0015 | .1285 | .06 | .07 |
| 4.00 | 2 | .0700 | .00000 | .00000 | .0700 | .0700 | .07 | .07 |
| 5.00 | 2 | .0750 | .00707 | .00500 | .0115 | .1385 | .07 | .08 |
| 6.00 | 2 | .0850 | .00707 | .00500 | .0215 | .1485 | .08 | .09 |
| 7.00 | 2 | .5350 | .00707 | .00500 | .4715 | .5985 | .53 | .54 |
| 8.00 | 2 | .5550 | .00707 | .00500 | .4915 | .6185 | .55 | .56 |
| 9.00 | 2 | .6800 | .07071 | .05000 | .0447 | 1.3153 | .63 | .73 |
| 10.00 | 2 | .6550 | .00707 | .00500 | .5915 | .7185 | .65 | .66 |
| Total | 20 | .3590 | .30861 | .06901 | .2146 | .5034 | .04 | .85 |

| **Multiple Comparisons** | | | | | | |
| --- | --- | --- | --- | --- | --- | --- |
| Dependent Variable: VAR00002 | | | | | | |
| Tukey HSD | | | | | | |
| (I) VAR00001 | (J) VAR00001 | Mean Difference (I-J) | Std. Error | Sig. | 95% Confidence Interval | |
|  |  |  |  |  | Lower Bound | Upper Bound |
| 1.00 | 2.00 | .78000^*^ | .02569 | .000 | .6783 | .8817 |
|  | 3.00 | .76000^*^ | .02569 | .000 | .6583 | .8617 |
|  | 4.00 | .75500^*^ | .02569 | .000 | .6533 | .8567 |
|  | 5.00 | .75000^*^ | .02569 | .000 | .6483 | .8517 |
|  | 6.00 | .74000^*^ | .02569 | .000 | .6383 | .8417 |
|  | 7.00 | .29000^*^ | .02569 | .000 | .1883 | .3917 |
|  | 8.00 | .27000^*^ | .02569 | .000 | .1683 | .3717 |
|  | 9.00 | .14500^*^ | .02569 | .005 | .0433 | .2467 |
|  | 10.00 | .17000^*^ | .02569 | .001 | .0683 | .2717 |
| 2.00 | 1.00 | -.78000^*^ | .02569 | .000 | -.8817 | -.6783 |
|  | 3.00 | -.02000 | .02569 | .997 | -.1217 | .0817 |
|  | 4.00 | -.02500 | .02569 | .988 | -.1267 | .0767 |
|  | 5.00 | -.03000 | .02569 | .962 | -.1317 | .0717 |
|  | 6.00 | -.04000 | .02569 | .842 | -.1417 | .0617 |
|  | 7.00 | -.49000^*^ | .02569 | .000 | -.5917 | -.3883 |
|  | 8.00 | -.51000^*^ | .02569 | .000 | -.6117 | -.4083 |
|  | 9.00 | -.63500^*^ | .02569 | .000 | -.7367 | -.5333 |
|  | 10.00 | -.61000^*^ | .02569 | .000 | -.7117 | -.5083 |
| 3.00 | 1.00 | -.76000^*^ | .02569 | .000 | -.8617 | -.6583 |
|  | 2.00 | .02000 | .02569 | .997 | -.0817 | .1217 |
|  | 4.00 | -.00500 | .02569 | 1.000 | -.1067 | .0967 |
|  | 5.00 | -.01000 | .02569 | 1.000 | -.1117 | .0917 |
|  | 6.00 | -.02000 | .02569 | .997 | -.1217 | .0817 |
|  | 7.00 | -.47000^*^ | .02569 | .000 | -.5717 | -.3683 |
|  | 8.00 | -.49000^*^ | .02569 | .000 | -.5917 | -.3883 |
|  | 9.00 | -.61500^*^ | .02569 | .000 | -.7167 | -.5133 |
|  | 10.00 | -.59000^*^ | .02569 | .000 | -.6917 | -.4883 |
| 4.00 | 1.00 | -.75500^*^ | .02569 | .000 | -.8567 | -.6533 |
|  | 2.00 | .02500 | .02569 | .988 | -.0767 | .1267 |
|  | 3.00 | .00500 | .02569 | 1.000 | -.0967 | .1067 |
|  | 5.00 | -.00500 | .02569 | 1.000 | -.1067 | .0967 |
|  | 6.00 | -.01500 | .02569 | 1.000 | -.1167 | .0867 |
|  | 7.00 | -.46500^*^ | .02569 | .000 | -.5667 | -.3633 |
|  | 8.00 | -.48500^*^ | .02569 | .000 | -.5867 | -.3833 |
|  | 9.00 | -.61000^*^ | .02569 | .000 | -.7117 | -.5083 |
|  | 10.00 | -.58500^*^ | .02569 | .000 | -.6867 | -.4833 |
| 5.00 | 1.00 | -.75000^*^ | .02569 | .000 | -.8517 | -.6483 |
|  | 2.00 | .03000 | .02569 | .962 | -.0717 | .1317 |
|  | 3.00 | .01000 | .02569 | 1.000 | -.0917 | .1117 |
|  | 4.00 | .00500 | .02569 | 1.000 | -.0967 | .1067 |
|  | 6.00 | -.01000 | .02569 | 1.000 | -.1117 | .0917 |
|  | 7.00 | -.46000^*^ | .02569 | .000 | -.5617 | -.3583 |
|  | 8.00 | -.48000^*^ | .02569 | .000 | -.5817 | -.3783 |
|  | 9.00 | -.60500^*^ | .02569 | .000 | -.7067 | -.5033 |
|  | 10.00 | -.58000^*^ | .02569 | .000 | -.6817 | -.4783 |
| 6.00 | 1.00 | -.74000^*^ | .02569 | .000 | -.8417 | -.6383 |
|  | 2.00 | .04000 | .02569 | .842 | -.0617 | .1417 |
|  | 3.00 | .02000 | .02569 | .997 | -.0817 | .1217 |
|  | 4.00 | .01500 | .02569 | 1.000 | -.0867 | .1167 |
|  | 5.00 | .01000 | .02569 | 1.000 | -.0917 | .1117 |
|  | 7.00 | -.45000^*^ | .02569 | .000 | -.5517 | -.3483 |
|  | 8.00 | -.47000^*^ | .02569 | .000 | -.5717 | -.3683 |
|  | 9.00 | -.59500^*^ | .02569 | .000 | -.6967 | -.4933 |
|  | 10.00 | -.57000^*^ | .02569 | .000 | -.6717 | -.4683 |
| 7.00 | 1.00 | -.29000^*^ | .02569 | .000 | -.3917 | -.1883 |
|  | 2.00 | .49000^*^ | .02569 | .000 | .3883 | .5917 |
|  | 3.00 | .47000^*^ | .02569 | .000 | .3683 | .5717 |
|  | 4.00 | .46500^*^ | .02569 | .000 | .3633 | .5667 |
|  | 5.00 | .46000^*^ | .02569 | .000 | .3583 | .5617 |
|  | 6.00 | .45000^*^ | .02569 | .000 | .3483 | .5517 |
|  | 8.00 | -.02000 | .02569 | .997 | -.1217 | .0817 |
|  | 9.00 | -.14500^*^ | .02569 | .005 | -.2467 | -.0433 |
|  | 10.00 | -.12000^*^ | .02569 | .018 | -.2217 | -.0183 |
| 8.00 | 1.00 | -.27000^*^ | .02569 | .000 | -.3717 | -.1683 |
|  | 2.00 | .51000^*^ | .02569 | .000 | .4083 | .6117 |
|  | 3.00 | .49000^*^ | .02569 | .000 | .3883 | .5917 |
|  | 4.00 | .48500^*^ | .02569 | .000 | .3833 | .5867 |
|  | 5.00 | .48000^*^ | .02569 | .000 | .3783 | .5817 |
|  | 6.00 | .47000^*^ | .02569 | .000 | .3683 | .5717 |
|  | 7.00 | .02000 | .02569 | .997 | -.0817 | .1217 |
|  | 9.00 | -.12500^*^ | .02569 | .014 | -.2267 | -.0233 |
|  | 10.00 | -.10000 | .02569 | .055 | -.2017 | .0017 |
| 9.00 | 1.00 | -.14500^*^ | .02569 | .005 | -.2467 | -.0433 |
|  | 2.00 | .63500^*^ | .02569 | .000 | .5333 | .7367 |
|  | 3.00 | .61500^*^ | .02569 | .000 | .5133 | .7167 |
|  | 4.00 | .61000^*^ | .02569 | .000 | .5083 | .7117 |
|  | 5.00 | .60500^*^ | .02569 | .000 | .5033 | .7067 |
|  | 6.00 | .59500^*^ | .02569 | .000 | .4933 | .6967 |
|  | 7.00 | .14500^*^ | .02569 | .005 | .0433 | .2467 |
|  | 8.00 | .12500^*^ | .02569 | .014 | .0233 | .2267 |
|  | 10.00 | .02500 | .02569 | .988 | -.0767 | .1267 |
| 10.00 | 1.00 | -.17000^*^ | .02569 | .001 | -.2717 | -.0683 |
|  | 2.00 | .61000^*^ | .02569 | .000 | .5083 | .7117 |
|  | 3.00 | .59000^*^ | .02569 | .000 | .4883 | .6917 |
|  | 4.00 | .58500^*^ | .02569 | .000 | .4833 | .6867 |
|  | 5.00 | .58000^*^ | .02569 | .000 | .4783 | .6817 |
|  | 6.00 | .57000^*^ | .02569 | .000 | .4683 | .6717 |
|  | 7.00 | .12000^*^ | .02569 | .018 | .0183 | .2217 |
|  | 8.00 | .10000 | .02569 | .055 | -.0017 | .2017 |
|  | 9.00 | -.02500 | .02569 | .988 | -.1267 | .0767 |
| *. The mean difference is significant at the 0.05 level. | | | | | | |
