# Supplementary material for: Efficacy of green synthesized silver nanoparticles via ginger rhizome extract against Leishmania major in vitro
Source: PLoS One. 2021 Aug 18;16(8):e0255571. doi: 10.1371/journal.pone.0255571 (PMC8372886; doi:10.1371/journal.pone.0255571)
Supplement: S3 Data — (DOCX) [file pone.0255571.s003.docx]

Count assay for parasite

Number 1: Control (witout treatment)

Number 2: treated with 40 µg/ml of nanoparticle

Number 3: treated with 20 µg/ml of nanoparticle

Number 4: treated with 10 µg/ml of nanoparticle

Number 5: treated with 5 µg/ml of nanoparticle

Number 6: treated with 2.5 µg/ml of nanoparticle

Number 7: treated with 1.25 µg/ml of nanoparticle

Number 8: treated with 0.625 µg/ml of nanoparticle

Number 9: treated with 0.312 µg/ml of nanoparticle

Number 10: Treated with Glucantime

Number 11: treated with amphotericin

| **Descriptives** | | | | | | | | | |
| --- | --- | --- | --- | --- | --- | --- | --- | --- | --- |
|  | | N | Mean | Std. Deviation | Std. Error | 95% Confidence Interval for Mean | | Minimum | Maximum |
|  |  |  |  |  |  | Lower Bound | Upper Bound |  |  |
| VAR00002 | 1.00 | 2 | 75.0000 | .00000 | .00000 | 75.0000 | 75.0000 | 75.00 | 75.00 |
|  | 2.00 | 2 | .0000 | .00000 | .00000 | .0000 | .0000 | .00 | .00 |
|  | 3.00 | 2 | .0000 | .00000 | .00000 | .0000 | .0000 | .00 | .00 |
|  | 4.00 | 2 | .0000 | .00000 | .00000 | .0000 | .0000 | .00 | .00 |
|  | 5.00 | 2 | 2.0000 | .00000 | .00000 | 2.0000 | 2.0000 | 2.00 | 2.00 |
|  | 6.00 | 2 | 25.5000 | .70711 | .50000 | 19.1469 | 31.8531 | 25.00 | 26.00 |
|  | 7.00 | 2 | 27.0000 | .00000 | .00000 | 27.0000 | 27.0000 | 27.00 | 27.00 |
|  | 8.00 | 2 | 28.5000 | .70711 | .50000 | 22.1469 | 34.8531 | 28.00 | 29.00 |
|  | 9.00 | 2 | 31.0000 | 1.41421 | 1.00000 | 18.2938 | 43.7062 | 30.00 | 32.00 |
|  | 10.00 | 2 | 20.0000 | 1.41421 | 1.00000 | 7.2938 | 32.7062 | 19.00 | 21.00 |
|  | 11.00 | 2 | 24.0000 | 1.41421 | 1.00000 | 11.2938 | 36.7062 | 23.00 | 25.00 |
|  | Total | 22 | 21.1818 | 21.43792 | 4.57058 | 11.6768 | 30.6869 | .00 | 75.00 |
| VAR00003 | 1.00 | 2 | 81.5000 | .70711 | .50000 | 75.1469 | 87.8531 | 81.00 | 82.00 |
|  | 2.00 | 2 | .0000 | .00000 | .00000 | .0000 | .0000 | .00 | .00 |
|  | 3.00 | 2 | .0000 | .00000 | .00000 | .0000 | .0000 | .00 | .00 |
|  | 4.00 | 2 | .0000 | .00000 | .00000 | .0000 | .0000 | .00 | .00 |
|  | 5.00 | 2 | .0000 | .00000 | .00000 | .0000 | .0000 | .00 | .00 |
|  | 6.00 | 2 | 26.0000 | 1.41421 | 1.00000 | 13.2938 | 38.7062 | 25.00 | 27.00 |
|  | 7.00 | 2 | 24.0000 | 1.41421 | 1.00000 | 11.2938 | 36.7062 | 23.00 | 25.00 |
|  | 8.00 | 2 | 25.0000 | 1.41421 | 1.00000 | 12.2938 | 37.7062 | 24.00 | 26.00 |
|  | 9.00 | 2 | 30.0000 | 1.41421 | 1.00000 | 17.2938 | 42.7062 | 29.00 | 31.00 |
|  | 10.00 | 2 | 18.0000 | 1.41421 | 1.00000 | 5.2938 | 30.7062 | 17.00 | 19.00 |
|  | 11.00 | 2 | 20.0000 | 1.41421 | 1.00000 | 7.2938 | 32.7062 | 19.00 | 21.00 |
|  | Total | 22 | 20.4091 | 23.02723 | 4.90942 | 10.1994 | 30.6188 | .00 | 82.00 |
| VAR00004 | 1.00 | 2 | 90.0000 | 1.41421 | 1.00000 | 77.2938 | 102.7062 | 89.00 | 91.00 |
|  | 2.00 | 2 | .0000 | .00000 | .00000 | .0000 | .0000 | .00 | .00 |
|  | 3.00 | 2 | .0000 | .00000 | .00000 | .0000 | .0000 | .00 | .00 |
|  | 4.00 | 2 | .0000 | .00000 | .00000 | .0000 | .0000 | .00 | .00 |
|  | 5.00 | 2 | .0000 | .00000 | .00000 | .0000 | .0000 | .00 | .00 |
|  | 6.00 | 2 | 12.0000 | 1.41421 | 1.00000 | -.7062 | 24.7062 | 11.00 | 13.00 |
|  | 7.00 | 2 | 18.0000 | 1.41421 | 1.00000 | 5.2938 | 30.7062 | 17.00 | 19.00 |
|  | 8.00 | 2 | 20.0000 | 1.41421 | 1.00000 | 7.2938 | 32.7062 | 19.00 | 21.00 |
|  | 9.00 | 2 | 21.0000 | 1.41421 | 1.00000 | 8.2938 | 33.7062 | 20.00 | 22.00 |
|  | 10.00 | 2 | 16.0000 | 1.41421 | 1.00000 | 3.2938 | 28.7062 | 15.00 | 17.00 |
|  | 11.00 | 2 | 14.0000 | 1.41421 | 1.00000 | 1.2938 | 26.7062 | 13.00 | 15.00 |
|  | Total | 22 | 17.3636 | 24.97913 | 5.32557 | 6.2885 | 28.4388 | .00 | 91.00 |

| **Multiple Comparisons** | | | | | | | |
| --- | --- | --- | --- | --- | --- | --- | --- |
| Tukey HSD | | | | | | | |
| Dependent Variable | (I) VAR00001 | (J) VAR00001 | Mean Difference (I-J) | Std. Error | Sig. | 95% Confidence Interval | |
|  |  |  |  |  |  | Lower Bound | Upper Bound |
| VAR00002 | 1.00 | 2.00 | 75.00000^*^ | .79772 | .000 | 71.8381 | 78.1619 |
|  |  | 3.00 | 75.00000^*^ | .79772 | .000 | 71.8381 | 78.1619 |
|  |  | 4.00 | 75.00000^*^ | .79772 | .000 | 71.8381 | 78.1619 |
|  |  | 5.00 | 73.00000^*^ | .79772 | .000 | 69.8381 | 76.1619 |
|  |  | 6.00 | 49.50000^*^ | .79772 | .000 | 46.3381 | 52.6619 |
|  |  | 7.00 | 48.00000^*^ | .79772 | .000 | 44.8381 | 51.1619 |
|  |  | 8.00 | 46.50000^*^ | .79772 | .000 | 43.3381 | 49.6619 |
|  |  | 9.00 | 44.00000^*^ | .79772 | .000 | 40.8381 | 47.1619 |
|  |  | 10.00 | 55.00000^*^ | .79772 | .000 | 51.8381 | 58.1619 |
|  |  | 11.00 | 51.00000^*^ | .79772 | .000 | 47.8381 | 54.1619 |
|  | 2.00 | 1.00 | -75.00000^*^ | .79772 | .000 | -78.1619 | -71.8381 |
|  |  | 3.00 | .00000 | .79772 | 1.000 | -3.1619 | 3.1619 |
|  |  | 4.00 | .00000 | .79772 | 1.000 | -3.1619 | 3.1619 |
|  |  | 5.00 | -2.00000 | .79772 | .388 | -5.1619 | 1.1619 |
|  |  | 6.00 | -25.50000^*^ | .79772 | .000 | -28.6619 | -22.3381 |
|  |  | 7.00 | -27.00000^*^ | .79772 | .000 | -30.1619 | -23.8381 |
|  |  | 8.00 | -28.50000^*^ | .79772 | .000 | -31.6619 | -25.3381 |
|  |  | 9.00 | -31.00000^*^ | .79772 | .000 | -34.1619 | -27.8381 |
|  |  | 10.00 | -20.00000^*^ | .79772 | .000 | -23.1619 | -16.8381 |
|  |  | 11.00 | -24.00000^*^ | .79772 | .000 | -27.1619 | -20.8381 |
|  | 3.00 | 1.00 | -75.00000^*^ | .79772 | .000 | -78.1619 | -71.8381 |
|  |  | 2.00 | .00000 | .79772 | 1.000 | -3.1619 | 3.1619 |
|  |  | 4.00 | .00000 | .79772 | 1.000 | -3.1619 | 3.1619 |
|  |  | 5.00 | -2.00000 | .79772 | .388 | -5.1619 | 1.1619 |
|  |  | 6.00 | -25.50000^*^ | .79772 | .000 | -28.6619 | -22.3381 |
|  |  | 7.00 | -27.00000^*^ | .79772 | .000 | -30.1619 | -23.8381 |
|  |  | 8.00 | -28.50000^*^ | .79772 | .000 | -31.6619 | -25.3381 |
|  |  | 9.00 | -31.00000^*^ | .79772 | .000 | -34.1619 | -27.8381 |
|  |  | 10.00 | -20.00000^*^ | .79772 | .000 | -23.1619 | -16.8381 |
|  |  | 11.00 | -24.00000^*^ | .79772 | .000 | -27.1619 | -20.8381 |
|  | 4.00 | 1.00 | -75.00000^*^ | .79772 | .000 | -78.1619 | -71.8381 |
|  |  | 2.00 | .00000 | .79772 | 1.000 | -3.1619 | 3.1619 |
|  |  | 3.00 | .00000 | .79772 | 1.000 | -3.1619 | 3.1619 |
|  |  | 5.00 | -2.00000 | .79772 | .388 | -5.1619 | 1.1619 |
|  |  | 6.00 | -25.50000^*^ | .79772 | .000 | -28.6619 | -22.3381 |
|  |  | 7.00 | -27.00000^*^ | .79772 | .000 | -30.1619 | -23.8381 |
|  |  | 8.00 | -28.50000^*^ | .79772 | .000 | -31.6619 | -25.3381 |
|  |  | 9.00 | -31.00000^*^ | .79772 | .000 | -34.1619 | -27.8381 |
|  |  | 10.00 | -20.00000^*^ | .79772 | .000 | -23.1619 | -16.8381 |
|  |  | 11.00 | -24.00000^*^ | .79772 | .000 | -27.1619 | -20.8381 |
|  | 5.00 | 1.00 | -73.00000^*^ | .79772 | .000 | -76.1619 | -69.8381 |
|  |  | 2.00 | 2.00000 | .79772 | .388 | -1.1619 | 5.1619 |
|  |  | 3.00 | 2.00000 | .79772 | .388 | -1.1619 | 5.1619 |
|  |  | 4.00 | 2.00000 | .79772 | .388 | -1.1619 | 5.1619 |
|  |  | 6.00 | -23.50000^*^ | .79772 | .000 | -26.6619 | -20.3381 |
|  |  | 7.00 | -25.00000^*^ | .79772 | .000 | -28.1619 | -21.8381 |
|  |  | 8.00 | -26.50000^*^ | .79772 | .000 | -29.6619 | -23.3381 |
|  |  | 9.00 | -29.00000^*^ | .79772 | .000 | -32.1619 | -25.8381 |
|  |  | 10.00 | -18.00000^*^ | .79772 | .000 | -21.1619 | -14.8381 |
|  |  | 11.00 | -22.00000^*^ | .79772 | .000 | -25.1619 | -18.8381 |
|  | 6.00 | 1.00 | -49.50000^*^ | .79772 | .000 | -52.6619 | -46.3381 |
|  |  | 2.00 | 25.50000^*^ | .79772 | .000 | 22.3381 | 28.6619 |
|  |  | 3.00 | 25.50000^*^ | .79772 | .000 | 22.3381 | 28.6619 |
|  |  | 4.00 | 25.50000^*^ | .79772 | .000 | 22.3381 | 28.6619 |
|  |  | 5.00 | 23.50000^*^ | .79772 | .000 | 20.3381 | 26.6619 |
|  |  | 7.00 | -1.50000 | .79772 | .719 | -4.6619 | 1.6619 |
|  |  | 8.00 | -3.00000 | .79772 | .068 | -6.1619 | .1619 |
|  |  | 9.00 | -5.50000^*^ | .79772 | .001 | -8.6619 | -2.3381 |
|  |  | 10.00 | 5.50000^*^ | .79772 | .001 | 2.3381 | 8.6619 |
|  |  | 11.00 | 1.50000 | .79772 | .719 | -1.6619 | 4.6619 |
|  | 7.00 | 1.00 | -48.00000^*^ | .79772 | .000 | -51.1619 | -44.8381 |
|  |  | 2.00 | 27.00000^*^ | .79772 | .000 | 23.8381 | 30.1619 |
|  |  | 3.00 | 27.00000^*^ | .79772 | .000 | 23.8381 | 30.1619 |
|  |  | 4.00 | 27.00000^*^ | .79772 | .000 | 23.8381 | 30.1619 |
|  |  | 5.00 | 25.00000^*^ | .79772 | .000 | 21.8381 | 28.1619 |
|  |  | 6.00 | 1.50000 | .79772 | .719 | -1.6619 | 4.6619 |
|  |  | 8.00 | -1.50000 | .79772 | .719 | -4.6619 | 1.6619 |
|  |  | 9.00 | -4.00000^*^ | .79772 | .010 | -7.1619 | -.8381 |
|  |  | 10.00 | 7.00000^*^ | .79772 | .000 | 3.8381 | 10.1619 |
|  |  | 11.00 | 3.00000 | .79772 | .068 | -.1619 | 6.1619 |
|  | 8.00 | 1.00 | -46.50000^*^ | .79772 | .000 | -49.6619 | -43.3381 |
|  |  | 2.00 | 28.50000^*^ | .79772 | .000 | 25.3381 | 31.6619 |
|  |  | 3.00 | 28.50000^*^ | .79772 | .000 | 25.3381 | 31.6619 |
|  |  | 4.00 | 28.50000^*^ | .79772 | .000 | 25.3381 | 31.6619 |
|  |  | 5.00 | 26.50000^*^ | .79772 | .000 | 23.3381 | 29.6619 |
|  |  | 6.00 | 3.00000 | .79772 | .068 | -.1619 | 6.1619 |
|  |  | 7.00 | 1.50000 | .79772 | .719 | -1.6619 | 4.6619 |
|  |  | 9.00 | -2.50000 | .79772 | .170 | -5.6619 | .6619 |
|  |  | 10.00 | 8.50000^*^ | .79772 | .000 | 5.3381 | 11.6619 |
|  |  | 11.00 | 4.50000^*^ | .79772 | .004 | 1.3381 | 7.6619 |
|  | 9.00 | 1.00 | -44.00000^*^ | .79772 | .000 | -47.1619 | -40.8381 |
|  |  | 2.00 | 31.00000^*^ | .79772 | .000 | 27.8381 | 34.1619 |
|  |  | 3.00 | 31.00000^*^ | .79772 | .000 | 27.8381 | 34.1619 |
|  |  | 4.00 | 31.00000^*^ | .79772 | .000 | 27.8381 | 34.1619 |
|  |  | 5.00 | 29.00000^*^ | .79772 | .000 | 25.8381 | 32.1619 |
|  |  | 6.00 | 5.50000^*^ | .79772 | .001 | 2.3381 | 8.6619 |
|  |  | 7.00 | 4.00000^*^ | .79772 | .010 | .8381 | 7.1619 |
|  |  | 8.00 | 2.50000 | .79772 | .170 | -.6619 | 5.6619 |
|  |  | 10.00 | 11.00000^*^ | .79772 | .000 | 7.8381 | 14.1619 |
|  |  | 11.00 | 7.00000^*^ | .79772 | .000 | 3.8381 | 10.1619 |
|  | 10.00 | 1.00 | -55.00000^*^ | .79772 | .000 | -58.1619 | -51.8381 |
|  |  | 2.00 | 20.00000^*^ | .79772 | .000 | 16.8381 | 23.1619 |
|  |  | 3.00 | 20.00000^*^ | .79772 | .000 | 16.8381 | 23.1619 |
|  |  | 4.00 | 20.00000^*^ | .79772 | .000 | 16.8381 | 23.1619 |
|  |  | 5.00 | 18.00000^*^ | .79772 | .000 | 14.8381 | 21.1619 |
|  |  | 6.00 | -5.50000^*^ | .79772 | .001 | -8.6619 | -2.3381 |
|  |  | 7.00 | -7.00000^*^ | .79772 | .000 | -10.1619 | -3.8381 |
|  |  | 8.00 | -8.50000^*^ | .79772 | .000 | -11.6619 | -5.3381 |
|  |  | 9.00 | -11.00000^*^ | .79772 | .000 | -14.1619 | -7.8381 |
|  |  | 11.00 | -4.00000^*^ | .79772 | .010 | -7.1619 | -.8381 |
|  | 11.00 | 1.00 | -51.00000^*^ | .79772 | .000 | -54.1619 | -47.8381 |
|  |  | 2.00 | 24.00000^*^ | .79772 | .000 | 20.8381 | 27.1619 |
|  |  | 3.00 | 24.00000^*^ | .79772 | .000 | 20.8381 | 27.1619 |
|  |  | 4.00 | 24.00000^*^ | .79772 | .000 | 20.8381 | 27.1619 |
|  |  | 5.00 | 22.00000^*^ | .79772 | .000 | 18.8381 | 25.1619 |
|  |  | 6.00 | -1.50000 | .79772 | .719 | -4.6619 | 1.6619 |
|  |  | 7.00 | -3.00000 | .79772 | .068 | -6.1619 | .1619 |
|  |  | 8.00 | -4.50000^*^ | .79772 | .004 | -7.6619 | -1.3381 |
|  |  | 9.00 | -7.00000^*^ | .79772 | .000 | -10.1619 | -3.8381 |
|  |  | 10.00 | 4.00000^*^ | .79772 | .010 | .8381 | 7.1619 |
| VAR00003 | 1.00 | 2.00 | 81.50000^*^ | 1.06600 | .000 | 77.2748 | 85.7252 |
|  |  | 3.00 | 81.50000^*^ | 1.06600 | .000 | 77.2748 | 85.7252 |
|  |  | 4.00 | 81.50000^*^ | 1.06600 | .000 | 77.2748 | 85.7252 |
|  |  | 5.00 | 81.50000^*^ | 1.06600 | .000 | 77.2748 | 85.7252 |
|  |  | 6.00 | 55.50000^*^ | 1.06600 | .000 | 51.2748 | 59.7252 |
|  |  | 7.00 | 57.50000^*^ | 1.06600 | .000 | 53.2748 | 61.7252 |
|  |  | 8.00 | 56.50000^*^ | 1.06600 | .000 | 52.2748 | 60.7252 |
|  |  | 9.00 | 51.50000^*^ | 1.06600 | .000 | 47.2748 | 55.7252 |
|  |  | 10.00 | 63.50000^*^ | 1.06600 | .000 | 59.2748 | 67.7252 |
|  |  | 11.00 | 61.50000^*^ | 1.06600 | .000 | 57.2748 | 65.7252 |
|  | 2.00 | 1.00 | -81.50000^*^ | 1.06600 | .000 | -85.7252 | -77.2748 |
|  |  | 3.00 | .00000 | 1.06600 | 1.000 | -4.2252 | 4.2252 |
|  |  | 4.00 | .00000 | 1.06600 | 1.000 | -4.2252 | 4.2252 |
|  |  | 5.00 | .00000 | 1.06600 | 1.000 | -4.2252 | 4.2252 |
|  |  | 6.00 | -26.00000^*^ | 1.06600 | .000 | -30.2252 | -21.7748 |
|  |  | 7.00 | -24.00000^*^ | 1.06600 | .000 | -28.2252 | -19.7748 |
|  |  | 8.00 | -25.00000^*^ | 1.06600 | .000 | -29.2252 | -20.7748 |
|  |  | 9.00 | -30.00000^*^ | 1.06600 | .000 | -34.2252 | -25.7748 |
|  |  | 10.00 | -18.00000^*^ | 1.06600 | .000 | -22.2252 | -13.7748 |
|  |  | 11.00 | -20.00000^*^ | 1.06600 | .000 | -24.2252 | -15.7748 |
|  | 3.00 | 1.00 | -81.50000^*^ | 1.06600 | .000 | -85.7252 | -77.2748 |
|  |  | 2.00 | .00000 | 1.06600 | 1.000 | -4.2252 | 4.2252 |
|  |  | 4.00 | .00000 | 1.06600 | 1.000 | -4.2252 | 4.2252 |
|  |  | 5.00 | .00000 | 1.06600 | 1.000 | -4.2252 | 4.2252 |
|  |  | 6.00 | -26.00000^*^ | 1.06600 | .000 | -30.2252 | -21.7748 |
|  |  | 7.00 | -24.00000^*^ | 1.06600 | .000 | -28.2252 | -19.7748 |
|  |  | 8.00 | -25.00000^*^ | 1.06600 | .000 | -29.2252 | -20.7748 |
|  |  | 9.00 | -30.00000^*^ | 1.06600 | .000 | -34.2252 | -25.7748 |
|  |  | 10.00 | -18.00000^*^ | 1.06600 | .000 | -22.2252 | -13.7748 |
|  |  | 11.00 | -20.00000^*^ | 1.06600 | .000 | -24.2252 | -15.7748 |
|  | 4.00 | 1.00 | -81.50000^*^ | 1.06600 | .000 | -85.7252 | -77.2748 |
|  |  | 2.00 | .00000 | 1.06600 | 1.000 | -4.2252 | 4.2252 |
|  |  | 3.00 | .00000 | 1.06600 | 1.000 | -4.2252 | 4.2252 |
|  |  | 5.00 | .00000 | 1.06600 | 1.000 | -4.2252 | 4.2252 |
|  |  | 6.00 | -26.00000^*^ | 1.06600 | .000 | -30.2252 | -21.7748 |
|  |  | 7.00 | -24.00000^*^ | 1.06600 | .000 | -28.2252 | -19.7748 |
|  |  | 8.00 | -25.00000^*^ | 1.06600 | .000 | -29.2252 | -20.7748 |
|  |  | 9.00 | -30.00000^*^ | 1.06600 | .000 | -34.2252 | -25.7748 |
|  |  | 10.00 | -18.00000^*^ | 1.06600 | .000 | -22.2252 | -13.7748 |
|  |  | 11.00 | -20.00000^*^ | 1.06600 | .000 | -24.2252 | -15.7748 |
|  | 5.00 | 1.00 | -81.50000^*^ | 1.06600 | .000 | -85.7252 | -77.2748 |
|  |  | 2.00 | .00000 | 1.06600 | 1.000 | -4.2252 | 4.2252 |
|  |  | 3.00 | .00000 | 1.06600 | 1.000 | -4.2252 | 4.2252 |
|  |  | 4.00 | .00000 | 1.06600 | 1.000 | -4.2252 | 4.2252 |
|  |  | 6.00 | -26.00000^*^ | 1.06600 | .000 | -30.2252 | -21.7748 |
|  |  | 7.00 | -24.00000^*^ | 1.06600 | .000 | -28.2252 | -19.7748 |
|  |  | 8.00 | -25.00000^*^ | 1.06600 | .000 | -29.2252 | -20.7748 |
|  |  | 9.00 | -30.00000^*^ | 1.06600 | .000 | -34.2252 | -25.7748 |
|  |  | 10.00 | -18.00000^*^ | 1.06600 | .000 | -22.2252 | -13.7748 |
|  |  | 11.00 | -20.00000^*^ | 1.06600 | .000 | -24.2252 | -15.7748 |
|  | 6.00 | 1.00 | -55.50000^*^ | 1.06600 | .000 | -59.7252 | -51.2748 |
|  |  | 2.00 | 26.00000^*^ | 1.06600 | .000 | 21.7748 | 30.2252 |
|  |  | 3.00 | 26.00000^*^ | 1.06600 | .000 | 21.7748 | 30.2252 |
|  |  | 4.00 | 26.00000^*^ | 1.06600 | .000 | 21.7748 | 30.2252 |
|  |  | 5.00 | 26.00000^*^ | 1.06600 | .000 | 21.7748 | 30.2252 |
|  |  | 7.00 | 2.00000 | 1.06600 | .721 | -2.2252 | 6.2252 |
|  |  | 8.00 | 1.00000 | 1.06600 | .994 | -3.2252 | 5.2252 |
|  |  | 9.00 | -4.00000 | 1.06600 | .069 | -8.2252 | .2252 |
|  |  | 10.00 | 8.00000^*^ | 1.06600 | .000 | 3.7748 | 12.2252 |
|  |  | 11.00 | 6.00000^*^ | 1.06600 | .004 | 1.7748 | 10.2252 |
|  | 7.00 | 1.00 | -57.50000^*^ | 1.06600 | .000 | -61.7252 | -53.2748 |
|  |  | 2.00 | 24.00000^*^ | 1.06600 | .000 | 19.7748 | 28.2252 |
|  |  | 3.00 | 24.00000^*^ | 1.06600 | .000 | 19.7748 | 28.2252 |
|  |  | 4.00 | 24.00000^*^ | 1.06600 | .000 | 19.7748 | 28.2252 |
|  |  | 5.00 | 24.00000^*^ | 1.06600 | .000 | 19.7748 | 28.2252 |
|  |  | 6.00 | -2.00000 | 1.06600 | .721 | -6.2252 | 2.2252 |
|  |  | 8.00 | -1.00000 | 1.06600 | .994 | -5.2252 | 3.2252 |
|  |  | 9.00 | -6.00000^*^ | 1.06600 | .004 | -10.2252 | -1.7748 |
|  |  | 10.00 | 6.00000^*^ | 1.06600 | .004 | 1.7748 | 10.2252 |
|  |  | 11.00 | 4.00000 | 1.06600 | .069 | -.2252 | 8.2252 |
|  | 8.00 | 1.00 | -56.50000^*^ | 1.06600 | .000 | -60.7252 | -52.2748 |
|  |  | 2.00 | 25.00000^*^ | 1.06600 | .000 | 20.7748 | 29.2252 |
|  |  | 3.00 | 25.00000^*^ | 1.06600 | .000 | 20.7748 | 29.2252 |
|  |  | 4.00 | 25.00000^*^ | 1.06600 | .000 | 20.7748 | 29.2252 |
|  |  | 5.00 | 25.00000^*^ | 1.06600 | .000 | 20.7748 | 29.2252 |
|  |  | 6.00 | -1.00000 | 1.06600 | .994 | -5.2252 | 3.2252 |
|  |  | 7.00 | 1.00000 | 1.06600 | .994 | -3.2252 | 5.2252 |
|  |  | 9.00 | -5.00000^*^ | 1.06600 | .017 | -9.2252 | -.7748 |
|  |  | 10.00 | 7.00000^*^ | 1.06600 | .001 | 2.7748 | 11.2252 |
|  |  | 11.00 | 5.00000^*^ | 1.06600 | .017 | .7748 | 9.2252 |
|  | 9.00 | 1.00 | -51.50000^*^ | 1.06600 | .000 | -55.7252 | -47.2748 |
|  |  | 2.00 | 30.00000^*^ | 1.06600 | .000 | 25.7748 | 34.2252 |
|  |  | 3.00 | 30.00000^*^ | 1.06600 | .000 | 25.7748 | 34.2252 |
|  |  | 4.00 | 30.00000^*^ | 1.06600 | .000 | 25.7748 | 34.2252 |
|  |  | 5.00 | 30.00000^*^ | 1.06600 | .000 | 25.7748 | 34.2252 |
|  |  | 6.00 | 4.00000 | 1.06600 | .069 | -.2252 | 8.2252 |
|  |  | 7.00 | 6.00000^*^ | 1.06600 | .004 | 1.7748 | 10.2252 |
|  |  | 8.00 | 5.00000^*^ | 1.06600 | .017 | .7748 | 9.2252 |
|  |  | 10.00 | 12.00000^*^ | 1.06600 | .000 | 7.7748 | 16.2252 |
|  |  | 11.00 | 10.00000^*^ | 1.06600 | .000 | 5.7748 | 14.2252 |
|  | 10.00 | 1.00 | -63.50000^*^ | 1.06600 | .000 | -67.7252 | -59.2748 |
|  |  | 2.00 | 18.00000^*^ | 1.06600 | .000 | 13.7748 | 22.2252 |
|  |  | 3.00 | 18.00000^*^ | 1.06600 | .000 | 13.7748 | 22.2252 |
|  |  | 4.00 | 18.00000^*^ | 1.06600 | .000 | 13.7748 | 22.2252 |
|  |  | 5.00 | 18.00000^*^ | 1.06600 | .000 | 13.7748 | 22.2252 |
|  |  | 6.00 | -8.00000^*^ | 1.06600 | .000 | -12.2252 | -3.7748 |
|  |  | 7.00 | -6.00000^*^ | 1.06600 | .004 | -10.2252 | -1.7748 |
|  |  | 8.00 | -7.00000^*^ | 1.06600 | .001 | -11.2252 | -2.7748 |
|  |  | 9.00 | -12.00000^*^ | 1.06600 | .000 | -16.2252 | -7.7748 |
|  |  | 11.00 | -2.00000 | 1.06600 | .721 | -6.2252 | 2.2252 |
|  | 11.00 | 1.00 | -61.50000^*^ | 1.06600 | .000 | -65.7252 | -57.2748 |
|  |  | 2.00 | 20.00000^*^ | 1.06600 | .000 | 15.7748 | 24.2252 |
|  |  | 3.00 | 20.00000^*^ | 1.06600 | .000 | 15.7748 | 24.2252 |
|  |  | 4.00 | 20.00000^*^ | 1.06600 | .000 | 15.7748 | 24.2252 |
|  |  | 5.00 | 20.00000^*^ | 1.06600 | .000 | 15.7748 | 24.2252 |
|  |  | 6.00 | -6.00000^*^ | 1.06600 | .004 | -10.2252 | -1.7748 |
|  |  | 7.00 | -4.00000 | 1.06600 | .069 | -8.2252 | .2252 |
|  |  | 8.00 | -5.00000^*^ | 1.06600 | .017 | -9.2252 | -.7748 |
|  |  | 9.00 | -10.00000^*^ | 1.06600 | .000 | -14.2252 | -5.7748 |
|  |  | 10.00 | 2.00000 | 1.06600 | .721 | -2.2252 | 6.2252 |
| VAR00004 | 1.00 | 2.00 | 90.00000^*^ | 1.12815 | .000 | 85.5284 | 94.4716 |
|  |  | 3.00 | 90.00000^*^ | 1.12815 | .000 | 85.5284 | 94.4716 |
|  |  | 4.00 | 90.00000^*^ | 1.12815 | .000 | 85.5284 | 94.4716 |
|  |  | 5.00 | 90.00000^*^ | 1.12815 | .000 | 85.5284 | 94.4716 |
|  |  | 6.00 | 78.00000^*^ | 1.12815 | .000 | 73.5284 | 82.4716 |
|  |  | 7.00 | 72.00000^*^ | 1.12815 | .000 | 67.5284 | 76.4716 |
|  |  | 8.00 | 70.00000^*^ | 1.12815 | .000 | 65.5284 | 74.4716 |
|  |  | 9.00 | 69.00000^*^ | 1.12815 | .000 | 64.5284 | 73.4716 |
|  |  | 10.00 | 74.00000^*^ | 1.12815 | .000 | 69.5284 | 78.4716 |
|  |  | 11.00 | 76.00000^*^ | 1.12815 | .000 | 71.5284 | 80.4716 |
|  | 2.00 | 1.00 | -90.00000^*^ | 1.12815 | .000 | -94.4716 | -85.5284 |
|  |  | 3.00 | .00000 | 1.12815 | 1.000 | -4.4716 | 4.4716 |
|  |  | 4.00 | .00000 | 1.12815 | 1.000 | -4.4716 | 4.4716 |
|  |  | 5.00 | .00000 | 1.12815 | 1.000 | -4.4716 | 4.4716 |
|  |  | 6.00 | -12.00000^*^ | 1.12815 | .000 | -16.4716 | -7.5284 |
|  |  | 7.00 | -18.00000^*^ | 1.12815 | .000 | -22.4716 | -13.5284 |
|  |  | 8.00 | -20.00000^*^ | 1.12815 | .000 | -24.4716 | -15.5284 |
|  |  | 9.00 | -21.00000^*^ | 1.12815 | .000 | -25.4716 | -16.5284 |
|  |  | 10.00 | -16.00000^*^ | 1.12815 | .000 | -20.4716 | -11.5284 |
|  |  | 11.00 | -14.00000^*^ | 1.12815 | .000 | -18.4716 | -9.5284 |
|  | 3.00 | 1.00 | -90.00000^*^ | 1.12815 | .000 | -94.4716 | -85.5284 |
|  |  | 2.00 | .00000 | 1.12815 | 1.000 | -4.4716 | 4.4716 |
|  |  | 4.00 | .00000 | 1.12815 | 1.000 | -4.4716 | 4.4716 |
|  |  | 5.00 | .00000 | 1.12815 | 1.000 | -4.4716 | 4.4716 |
|  |  | 6.00 | -12.00000^*^ | 1.12815 | .000 | -16.4716 | -7.5284 |
|  |  | 7.00 | -18.00000^*^ | 1.12815 | .000 | -22.4716 | -13.5284 |
|  |  | 8.00 | -20.00000^*^ | 1.12815 | .000 | -24.4716 | -15.5284 |
|  |  | 9.00 | -21.00000^*^ | 1.12815 | .000 | -25.4716 | -16.5284 |
|  |  | 10.00 | -16.00000^*^ | 1.12815 | .000 | -20.4716 | -11.5284 |
|  |  | 11.00 | -14.00000^*^ | 1.12815 | .000 | -18.4716 | -9.5284 |
|  | 4.00 | 1.00 | -90.00000^*^ | 1.12815 | .000 | -94.4716 | -85.5284 |
|  |  | 2.00 | .00000 | 1.12815 | 1.000 | -4.4716 | 4.4716 |
|  |  | 3.00 | .00000 | 1.12815 | 1.000 | -4.4716 | 4.4716 |
|  |  | 5.00 | .00000 | 1.12815 | 1.000 | -4.4716 | 4.4716 |
|  |  | 6.00 | -12.00000^*^ | 1.12815 | .000 | -16.4716 | -7.5284 |
|  |  | 7.00 | -18.00000^*^ | 1.12815 | .000 | -22.4716 | -13.5284 |
|  |  | 8.00 | -20.00000^*^ | 1.12815 | .000 | -24.4716 | -15.5284 |
|  |  | 9.00 | -21.00000^*^ | 1.12815 | .000 | -25.4716 | -16.5284 |
|  |  | 10.00 | -16.00000^*^ | 1.12815 | .000 | -20.4716 | -11.5284 |
|  |  | 11.00 | -14.00000^*^ | 1.12815 | .000 | -18.4716 | -9.5284 |
|  | 5.00 | 1.00 | -90.00000^*^ | 1.12815 | .000 | -94.4716 | -85.5284 |
|  |  | 2.00 | .00000 | 1.12815 | 1.000 | -4.4716 | 4.4716 |
|  |  | 3.00 | .00000 | 1.12815 | 1.000 | -4.4716 | 4.4716 |
|  |  | 4.00 | .00000 | 1.12815 | 1.000 | -4.4716 | 4.4716 |
|  |  | 6.00 | -12.00000^*^ | 1.12815 | .000 | -16.4716 | -7.5284 |
|  |  | 7.00 | -18.00000^*^ | 1.12815 | .000 | -22.4716 | -13.5284 |
|  |  | 8.00 | -20.00000^*^ | 1.12815 | .000 | -24.4716 | -15.5284 |
|  |  | 9.00 | -21.00000^*^ | 1.12815 | .000 | -25.4716 | -16.5284 |
|  |  | 10.00 | -16.00000^*^ | 1.12815 | .000 | -20.4716 | -11.5284 |
|  |  | 11.00 | -14.00000^*^ | 1.12815 | .000 | -18.4716 | -9.5284 |
|  | 6.00 | 1.00 | -78.00000^*^ | 1.12815 | .000 | -82.4716 | -73.5284 |
|  |  | 2.00 | 12.00000^*^ | 1.12815 | .000 | 7.5284 | 16.4716 |
|  |  | 3.00 | 12.00000^*^ | 1.12815 | .000 | 7.5284 | 16.4716 |
|  |  | 4.00 | 12.00000^*^ | 1.12815 | .000 | 7.5284 | 16.4716 |
|  |  | 5.00 | 12.00000^*^ | 1.12815 | .000 | 7.5284 | 16.4716 |
|  |  | 7.00 | -6.00000^*^ | 1.12815 | .007 | -10.4716 | -1.5284 |
|  |  | 8.00 | -8.00000^*^ | 1.12815 | .001 | -12.4716 | -3.5284 |
|  |  | 9.00 | -9.00000^*^ | 1.12815 | .000 | -13.4716 | -4.5284 |
|  |  | 10.00 | -4.00000 | 1.12815 | .094 | -8.4716 | .4716 |
|  |  | 11.00 | -2.00000 | 1.12815 | .774 | -6.4716 | 2.4716 |
|  | 7.00 | 1.00 | -72.00000^*^ | 1.12815 | .000 | -76.4716 | -67.5284 |
|  |  | 2.00 | 18.00000^*^ | 1.12815 | .000 | 13.5284 | 22.4716 |
|  |  | 3.00 | 18.00000^*^ | 1.12815 | .000 | 13.5284 | 22.4716 |
|  |  | 4.00 | 18.00000^*^ | 1.12815 | .000 | 13.5284 | 22.4716 |
|  |  | 5.00 | 18.00000^*^ | 1.12815 | .000 | 13.5284 | 22.4716 |
|  |  | 6.00 | 6.00000^*^ | 1.12815 | .007 | 1.5284 | 10.4716 |
|  |  | 8.00 | -2.00000 | 1.12815 | .774 | -6.4716 | 2.4716 |
|  |  | 9.00 | -3.00000 | 1.12815 | .322 | -7.4716 | 1.4716 |
|  |  | 10.00 | 2.00000 | 1.12815 | .774 | -2.4716 | 6.4716 |
|  |  | 11.00 | 4.00000 | 1.12815 | .094 | -.4716 | 8.4716 |
|  | 8.00 | 1.00 | -70.00000^*^ | 1.12815 | .000 | -74.4716 | -65.5284 |
|  |  | 2.00 | 20.00000^*^ | 1.12815 | .000 | 15.5284 | 24.4716 |
|  |  | 3.00 | 20.00000^*^ | 1.12815 | .000 | 15.5284 | 24.4716 |
|  |  | 4.00 | 20.00000^*^ | 1.12815 | .000 | 15.5284 | 24.4716 |
|  |  | 5.00 | 20.00000^*^ | 1.12815 | .000 | 15.5284 | 24.4716 |
|  |  | 6.00 | 8.00000^*^ | 1.12815 | .001 | 3.5284 | 12.4716 |
|  |  | 7.00 | 2.00000 | 1.12815 | .774 | -2.4716 | 6.4716 |
|  |  | 9.00 | -1.00000 | 1.12815 | .996 | -5.4716 | 3.4716 |
|  |  | 10.00 | 4.00000 | 1.12815 | .094 | -.4716 | 8.4716 |
|  |  | 11.00 | 6.00000^*^ | 1.12815 | .007 | 1.5284 | 10.4716 |
|  | 9.00 | 1.00 | -69.00000^*^ | 1.12815 | .000 | -73.4716 | -64.5284 |
|  |  | 2.00 | 21.00000^*^ | 1.12815 | .000 | 16.5284 | 25.4716 |
|  |  | 3.00 | 21.00000^*^ | 1.12815 | .000 | 16.5284 | 25.4716 |
|  |  | 4.00 | 21.00000^*^ | 1.12815 | .000 | 16.5284 | 25.4716 |
|  |  | 5.00 | 21.00000^*^ | 1.12815 | .000 | 16.5284 | 25.4716 |
|  |  | 6.00 | 9.00000^*^ | 1.12815 | .000 | 4.5284 | 13.4716 |
|  |  | 7.00 | 3.00000 | 1.12815 | .322 | -1.4716 | 7.4716 |
|  |  | 8.00 | 1.00000 | 1.12815 | .996 | -3.4716 | 5.4716 |
|  |  | 10.00 | 5.00000^*^ | 1.12815 | .025 | .5284 | 9.4716 |
|  |  | 11.00 | 7.00000^*^ | 1.12815 | .002 | 2.5284 | 11.4716 |
|  | 10.00 | 1.00 | -74.00000^*^ | 1.12815 | .000 | -78.4716 | -69.5284 |
|  |  | 2.00 | 16.00000^*^ | 1.12815 | .000 | 11.5284 | 20.4716 |
|  |  | 3.00 | 16.00000^*^ | 1.12815 | .000 | 11.5284 | 20.4716 |
|  |  | 4.00 | 16.00000^*^ | 1.12815 | .000 | 11.5284 | 20.4716 |
|  |  | 5.00 | 16.00000^*^ | 1.12815 | .000 | 11.5284 | 20.4716 |
|  |  | 6.00 | 4.00000 | 1.12815 | .094 | -.4716 | 8.4716 |
|  |  | 7.00 | -2.00000 | 1.12815 | .774 | -6.4716 | 2.4716 |
|  |  | 8.00 | -4.00000 | 1.12815 | .094 | -8.4716 | .4716 |
|  |  | 9.00 | -5.00000^*^ | 1.12815 | .025 | -9.4716 | -.5284 |
|  |  | 11.00 | 2.00000 | 1.12815 | .774 | -2.4716 | 6.4716 |
|  | 11.00 | 1.00 | -76.00000^*^ | 1.12815 | .000 | -80.4716 | -71.5284 |
|  |  | 2.00 | 14.00000^*^ | 1.12815 | .000 | 9.5284 | 18.4716 |
|  |  | 3.00 | 14.00000^*^ | 1.12815 | .000 | 9.5284 | 18.4716 |
|  |  | 4.00 | 14.00000^*^ | 1.12815 | .000 | 9.5284 | 18.4716 |
|  |  | 5.00 | 14.00000^*^ | 1.12815 | .000 | 9.5284 | 18.4716 |
|  |  | 6.00 | 2.00000 | 1.12815 | .774 | -2.4716 | 6.4716 |
|  |  | 7.00 | -4.00000 | 1.12815 | .094 | -8.4716 | .4716 |
|  |  | 8.00 | -6.00000^*^ | 1.12815 | .007 | -10.4716 | -1.5284 |
|  |  | 9.00 | -7.00000^*^ | 1.12815 | .002 | -11.4716 | -2.5284 |
|  |  | 10.00 | -2.00000 | 1.12815 | .774 | -6.4716 | 2.4716 |
| *. The mean difference is significant at the 0.05 level. | | | | | | | |
