# Supplementary material for: Efficacy of green synthesized silver nanoparticles via ginger rhizome extract against Leishmania major in vitro
Source: PLoS One. 2021 Aug 18;16(8):e0255571. doi: 10.1371/journal.pone.0255571 (PMC8372886; doi:10.1371/journal.pone.0255571)
Supplement: S4 Data — (DOCX) [file pone.0255571.s004.docx]

MTT Assay for parasite

Number 1: Control (witout treatment)

Number 2: treated with 40 µg/ml of nanoparticle

Number 3: treated with 20 µg/ml of nanoparticle

Number 4: treated with 10 µg/ml of nanoparticle

Number 5: treated with 5 µg/ml of nanoparticle

Number 6: treated with 2.5 µg/ml of nanoparticle

Number 7: treated with 1.25 µg/ml of nanoparticle

Number 8: treated with 0.625 µg/ml of nanoparticle

Number 9: treated with 0.312 µg/ml of nanoparticle

Number 10: Treated with Glucantime

Number 11: treated with amphotericin

| **Descriptives** | | | | | | | | |
| --- | --- | --- | --- | --- | --- | --- | --- | --- |
| VAR00002 | | | | | | | | |
|  | N | Mean | Std. Deviation | Std. Error | 95% Confidence Interval for Mean | | Minimum | Maximum |
|  |  |  |  |  | Lower Bound | Upper Bound |  |  |
| 1.00 | 2 | .5150 | .00707 | .00500 | .4515 | .5785 | .51 | .52 |
| 2.00 | 2 | .1450 | .00707 | .00500 | .0815 | .2085 | .14 | .15 |
| 3.00 | 2 | .1600 | .00000 | .00000 | .1600 | .1600 | .16 | .16 |
| 4.00 | 2 | .2150 | .00707 | .00500 | .1515 | .2785 | .21 | .22 |
| 5.00 | 2 | .2050 | .00707 | .00500 | .1415 | .2685 | .20 | .21 |
| 6.00 | 2 | .2250 | .00707 | .00500 | .1615 | .2885 | .22 | .23 |
| 7.00 | 2 | .2750 | .00707 | .00500 | .2115 | .3385 | .27 | .28 |
| 8.00 | 2 | .3150 | .00707 | .00500 | .2515 | .3785 | .31 | .32 |
| 9.00 | 2 | .3650 | .00707 | .00500 | .3015 | .4285 | .36 | .37 |
| 10.00 | 2 | .3550 | .00707 | .00500 | .2915 | .4185 | .35 | .36 |
| 11.00 | 2 | .3750 | .00707 | .00500 | .3115 | .4385 | .37 | .38 |
| Total | 22 | .2864 | .10852 | .02314 | .2382 | .3345 | .14 | .52 |

| **Multiple Comparisons** | | | | | | |
| --- | --- | --- | --- | --- | --- | --- |
| Dependent Variable: VAR00002 | | | | | | |
| Tukey HSD | | | | | | |
| (I) VAR00001 | (J) VAR00001 | Mean Difference (I-J) | Std. Error | Sig. | 95% Confidence Interval | |
|  |  |  |  |  | Lower Bound | Upper Bound |
| 1.00 | 2.00 | .37000^*^ | .00674 | .000 | .3433 | .3967 |
|  | 3.00 | .35500^*^ | .00674 | .000 | .3283 | .3817 |
|  | 4.00 | .30000^*^ | .00674 | .000 | .2733 | .3267 |
|  | 5.00 | .31000^*^ | .00674 | .000 | .2833 | .3367 |
|  | 6.00 | .29000^*^ | .00674 | .000 | .2633 | .3167 |
|  | 7.00 | .24000^*^ | .00674 | .000 | .2133 | .2667 |
|  | 8.00 | .20000^*^ | .00674 | .000 | .1733 | .2267 |
|  | 9.00 | .15000^*^ | .00674 | .000 | .1233 | .1767 |
|  | 10.00 | .16000^*^ | .00674 | .000 | .1333 | .1867 |
|  | 11.00 | .14000^*^ | .00674 | .000 | .1133 | .1667 |
| 2.00 | 1.00 | -.37000^*^ | .00674 | .000 | -.3967 | -.3433 |
|  | 3.00 | -.01500 | .00674 | .530 | -.0417 | .0117 |
|  | 4.00 | -.07000^*^ | .00674 | .000 | -.0967 | -.0433 |
|  | 5.00 | -.06000^*^ | .00674 | .000 | -.0867 | -.0333 |
|  | 6.00 | -.08000^*^ | .00674 | .000 | -.1067 | -.0533 |
|  | 7.00 | -.13000^*^ | .00674 | .000 | -.1567 | -.1033 |
|  | 8.00 | -.17000^*^ | .00674 | .000 | -.1967 | -.1433 |
|  | 9.00 | -.22000^*^ | .00674 | .000 | -.2467 | -.1933 |
|  | 10.00 | -.21000^*^ | .00674 | .000 | -.2367 | -.1833 |
|  | 11.00 | -.23000^*^ | .00674 | .000 | -.2567 | -.2033 |
| 3.00 | 1.00 | -.35500^*^ | .00674 | .000 | -.3817 | -.3283 |
|  | 2.00 | .01500 | .00674 | .530 | -.0117 | .0417 |
|  | 4.00 | -.05500^*^ | .00674 | .000 | -.0817 | -.0283 |
|  | 5.00 | -.04500^*^ | .00674 | .001 | -.0717 | -.0183 |
|  | 6.00 | -.06500^*^ | .00674 | .000 | -.0917 | -.0383 |
|  | 7.00 | -.11500^*^ | .00674 | .000 | -.1417 | -.0883 |
|  | 8.00 | -.15500^*^ | .00674 | .000 | -.1817 | -.1283 |
|  | 9.00 | -.20500^*^ | .00674 | .000 | -.2317 | -.1783 |
|  | 10.00 | -.19500^*^ | .00674 | .000 | -.2217 | -.1683 |
|  | 11.00 | -.21500^*^ | .00674 | .000 | -.2417 | -.1883 |
| 4.00 | 1.00 | -.30000^*^ | .00674 | .000 | -.3267 | -.2733 |
|  | 2.00 | .07000^*^ | .00674 | .000 | .0433 | .0967 |
|  | 3.00 | .05500^*^ | .00674 | .000 | .0283 | .0817 |
|  | 5.00 | .01000 | .00674 | .899 | -.0167 | .0367 |
|  | 6.00 | -.01000 | .00674 | .899 | -.0367 | .0167 |
|  | 7.00 | -.06000^*^ | .00674 | .000 | -.0867 | -.0333 |
|  | 8.00 | -.10000^*^ | .00674 | .000 | -.1267 | -.0733 |
|  | 9.00 | -.15000^*^ | .00674 | .000 | -.1767 | -.1233 |
|  | 10.00 | -.14000^*^ | .00674 | .000 | -.1667 | -.1133 |
|  | 11.00 | -.16000^*^ | .00674 | .000 | -.1867 | -.1333 |
| 5.00 | 1.00 | -.31000^*^ | .00674 | .000 | -.3367 | -.2833 |
|  | 2.00 | .06000^*^ | .00674 | .000 | .0333 | .0867 |
|  | 3.00 | .04500^*^ | .00674 | .001 | .0183 | .0717 |
|  | 4.00 | -.01000 | .00674 | .899 | -.0367 | .0167 |
|  | 6.00 | -.02000 | .00674 | .215 | -.0467 | .0067 |
|  | 7.00 | -.07000^*^ | .00674 | .000 | -.0967 | -.0433 |
|  | 8.00 | -.11000^*^ | .00674 | .000 | -.1367 | -.0833 |
|  | 9.00 | -.16000^*^ | .00674 | .000 | -.1867 | -.1333 |
|  | 10.00 | -.15000^*^ | .00674 | .000 | -.1767 | -.1233 |
|  | 11.00 | -.17000^*^ | .00674 | .000 | -.1967 | -.1433 |
| 6.00 | 1.00 | -.29000^*^ | .00674 | .000 | -.3167 | -.2633 |
|  | 2.00 | .08000^*^ | .00674 | .000 | .0533 | .1067 |
|  | 3.00 | .06500^*^ | .00674 | .000 | .0383 | .0917 |
|  | 4.00 | .01000 | .00674 | .899 | -.0167 | .0367 |
|  | 5.00 | .02000 | .00674 | .215 | -.0067 | .0467 |
|  | 7.00 | -.05000^*^ | .00674 | .000 | -.0767 | -.0233 |
|  | 8.00 | -.09000^*^ | .00674 | .000 | -.1167 | -.0633 |
|  | 9.00 | -.14000^*^ | .00674 | .000 | -.1667 | -.1133 |
|  | 10.00 | -.13000^*^ | .00674 | .000 | -.1567 | -.1033 |
|  | 11.00 | -.15000^*^ | .00674 | .000 | -.1767 | -.1233 |
| 7.00 | 1.00 | -.24000^*^ | .00674 | .000 | -.2667 | -.2133 |
|  | 2.00 | .13000^*^ | .00674 | .000 | .1033 | .1567 |
|  | 3.00 | .11500^*^ | .00674 | .000 | .0883 | .1417 |
|  | 4.00 | .06000^*^ | .00674 | .000 | .0333 | .0867 |
|  | 5.00 | .07000^*^ | .00674 | .000 | .0433 | .0967 |
|  | 6.00 | .05000^*^ | .00674 | .000 | .0233 | .0767 |
|  | 8.00 | -.04000^*^ | .00674 | .003 | -.0667 | -.0133 |
|  | 9.00 | -.09000^*^ | .00674 | .000 | -.1167 | -.0633 |
|  | 10.00 | -.08000^*^ | .00674 | .000 | -.1067 | -.0533 |
|  | 11.00 | -.10000^*^ | .00674 | .000 | -.1267 | -.0733 |
| 8.00 | 1.00 | -.20000^*^ | .00674 | .000 | -.2267 | -.1733 |
|  | 2.00 | .17000^*^ | .00674 | .000 | .1433 | .1967 |
|  | 3.00 | .15500^*^ | .00674 | .000 | .1283 | .1817 |
|  | 4.00 | .10000^*^ | .00674 | .000 | .0733 | .1267 |
|  | 5.00 | .11000^*^ | .00674 | .000 | .0833 | .1367 |
|  | 6.00 | .09000^*^ | .00674 | .000 | .0633 | .1167 |
|  | 7.00 | .04000^*^ | .00674 | .003 | .0133 | .0667 |
|  | 9.00 | -.05000^*^ | .00674 | .000 | -.0767 | -.0233 |
|  | 10.00 | -.04000^*^ | .00674 | .003 | -.0667 | -.0133 |
|  | 11.00 | -.06000^*^ | .00674 | .000 | -.0867 | -.0333 |
| 9.00 | 1.00 | -.15000^*^ | .00674 | .000 | -.1767 | -.1233 |
|  | 2.00 | .22000^*^ | .00674 | .000 | .1933 | .2467 |
|  | 3.00 | .20500^*^ | .00674 | .000 | .1783 | .2317 |
|  | 4.00 | .15000^*^ | .00674 | .000 | .1233 | .1767 |
|  | 5.00 | .16000^*^ | .00674 | .000 | .1333 | .1867 |
|  | 6.00 | .14000^*^ | .00674 | .000 | .1133 | .1667 |
|  | 7.00 | .09000^*^ | .00674 | .000 | .0633 | .1167 |
|  | 8.00 | .05000^*^ | .00674 | .000 | .0233 | .0767 |
|  | 10.00 | .01000 | .00674 | .899 | -.0167 | .0367 |
|  | 11.00 | -.01000 | .00674 | .899 | -.0367 | .0167 |
| 10.00 | 1.00 | -.16000^*^ | .00674 | .000 | -.1867 | -.1333 |
|  | 2.00 | .21000^*^ | .00674 | .000 | .1833 | .2367 |
|  | 3.00 | .19500^*^ | .00674 | .000 | .1683 | .2217 |
|  | 4.00 | .14000^*^ | .00674 | .000 | .1133 | .1667 |
|  | 5.00 | .15000^*^ | .00674 | .000 | .1233 | .1767 |
|  | 6.00 | .13000^*^ | .00674 | .000 | .1033 | .1567 |
|  | 7.00 | .08000^*^ | .00674 | .000 | .0533 | .1067 |
|  | 8.00 | .04000^*^ | .00674 | .003 | .0133 | .0667 |
|  | 9.00 | -.01000 | .00674 | .899 | -.0367 | .0167 |
|  | 11.00 | -.02000 | .00674 | .215 | -.0467 | .0067 |
| 11.00 | 1.00 | -.14000^*^ | .00674 | .000 | -.1667 | -.1133 |
|  | 2.00 | .23000^*^ | .00674 | .000 | .2033 | .2567 |
|  | 3.00 | .21500^*^ | .00674 | .000 | .1883 | .2417 |
|  | 4.00 | .16000^*^ | .00674 | .000 | .1333 | .1867 |
|  | 5.00 | .17000^*^ | .00674 | .000 | .1433 | .1967 |
|  | 6.00 | .15000^*^ | .00674 | .000 | .1233 | .1767 |
|  | 7.00 | .10000^*^ | .00674 | .000 | .0733 | .1267 |
|  | 8.00 | .06000^*^ | .00674 | .000 | .0333 | .0867 |
|  | 9.00 | .01000 | .00674 | .899 | -.0167 | .0367 |
|  | 10.00 | .02000 | .00674 | .215 | -.0067 | .0467 |
| *. The mean difference is significant at the 0.05 level. | | | | | | |
